# Sequential state discrimination of coherent states

**DOI:** 10.1038/s41598-018-35047-6

**Published:** 2018-11-16

**Authors:** Min Namkung, Younghun Kwon

**Affiliations:** Department of Applied Physics, Hanyang University, Ansan, Kyunggi-Do 425-791 South Korea

## Abstract

Sequential state discrimination is a strategy for quantum state discrimination of a sender’s quantum states when N receivers are separately located. In this report, we propose optical designs that can perform sequential state discrimination of two coherent states. For this purpose, we consider not only binary phase-shifting-key (BPSK) signals but also general coherent states, with arbitrary prior probabilities. Since our optical designs do not include electric feedback, they can be implemented without difficulty. Furthermore, we analyze our proposal for the case of photon loss. We also demonstrate that sequential state discrimination of two coherent states performs better than the probabilistic quantum cloning strategy. This proposal can facilitate multiparty QKD based on coherent states.

## Introduction

In quantum physics, if quantum states of a physical system are not orthogonal to each other, it is not always possible to determine the quantum state of the system. Therefore, to discriminate the quantum state, it is necessary to establish an appropriate strategy. Quantum state discrimination is an important research topics in quantum information processing. This concept can be understood as a game between a sender Alice and a receiver Bob. Alice prepares a quantum state out of *N* quantum states, with a prior probability. The prior probability is known to the sender and the receiver. Bob sets up his measurement system to optimaly discriminate the quantum states of Alice. The measurement result of Bob is divided into an inconclusive result and *N* conclusive results. When Bob obtains an inconclusive result, he cannot guess the quantum state sent by Alice. If Bob has a conclusive result, he can guess the quantum state prepared by Alice. However, every conclusive result does not produce a correct result.

Therefore, quantum state discrimination requires a suitable strategy. When only conclusive results are allowed, one possible strategy is to maximize Bob’s guessing probability. This strategy is called minimum error discrimination^1–5^. Meanwhile, when the quantum states of Alice are linearly independent, it is possible for Bob to develop a strategy whereby he can trust his conclusive result^6,7^. This strategy is called unambiguous discrimination^8–12^, which can be applied to not only quantum key distribution (QKD)^13^ but also quantum state tomography^14^. The maximal confidence strategy^15^ is one where Bob sets up a measurement and maximizes the confidence of the conclusive result. In the case of error margin strategy, there is a finite margin of error probability, and Bob minimizes the possibility of inconclusive result^16–19^. The strategy of fixed rate of inconclusive result^20–25^ was proposed whereby the probability an inconclusive result is fixed.

In 2013, J. A. Bergou *et al*.^26^ proposed *sequential state discrimination*, which can be applied to multiple receivers. Let us assume that a sender Alice, and two receivers Bob and Charlie are separated in space. In sequential state discrimination, Bob performs non-optimal unambiguous discrimination on the quantum states of Alice. If he obtains a conclusive result, he sends his post-measurement state to Charlie. Charlie then performs optimal unambiguous discrimination on the post-measurement state of Bob. It should be noted that classical communication is not allowed between Bob and Charlie. The purpose of sequential state discrimination is to maximize the probability that Bob and Charlie correctly discriminate the quantum states of Alice. J. A. Bergou *et al*.^26^ and C.-Q. Pang *et al*.^27^ provided the optimal success probability of sequential state discrimination for two pure states with identical prior probability. In 2017, sequential state discrimination of two mixed states was investigated^28^. Also, M. Hillery and J. Mimih^29^ produced the success probability of sequential state discrimination for *N* symmetric pure states. In 2018, the structure of sequential state discrimination for arbitrary *N* pure states with general prior probability was investigated^30^. In fact, sequential state discrimination provides the answer to the question “Can we obtain information about a before-measurement quantum state, from a post-measurement state?”^31^. In addition, sequential state discrimination can be used for sharing a secret key in mult-parties.

Recently, M. A. Solis-Prosser *et al*.^32^ developed an optical design for sequential state discrimination of non-orthogonal two polarized photon states. In this report, we propose optical designs for sequential state discrimination of two arbitrary coherent states, which can produce an optimal success probability. Our models are based on the result of K. Banaszek^33^ and B. Huttner *et al*.^34^, which will be called *Banaszek model* and *Huttner*-*like model*. Even though our models can optimally perform sequential state discrimination of arbitrary two coherent states, they do not require electric feedback^35–39^ or interactions with other systems^40,41^. Our models consist only of a beam combiner, beam splitter, and photon detector. We can show that by using the Banaszek model or the Huttner-like model, Bob and Charlie can achieve the optimal success probability. It should be noted that since the Huttner-like model uses a mixture of auxiliary coherent light, it may be more applicable to secure QKD. Furthermore, we demonstrate an analysis of sequential state discrimination of two coherent states, in a realistic situation involving photon loss. In addition, we show that sequential state discrimination of two coherent states can perform better than probabilistic quantum cloning^42^.

## Results

### Optimal sequential state discrimination of binary pure states

**Figure 1:**
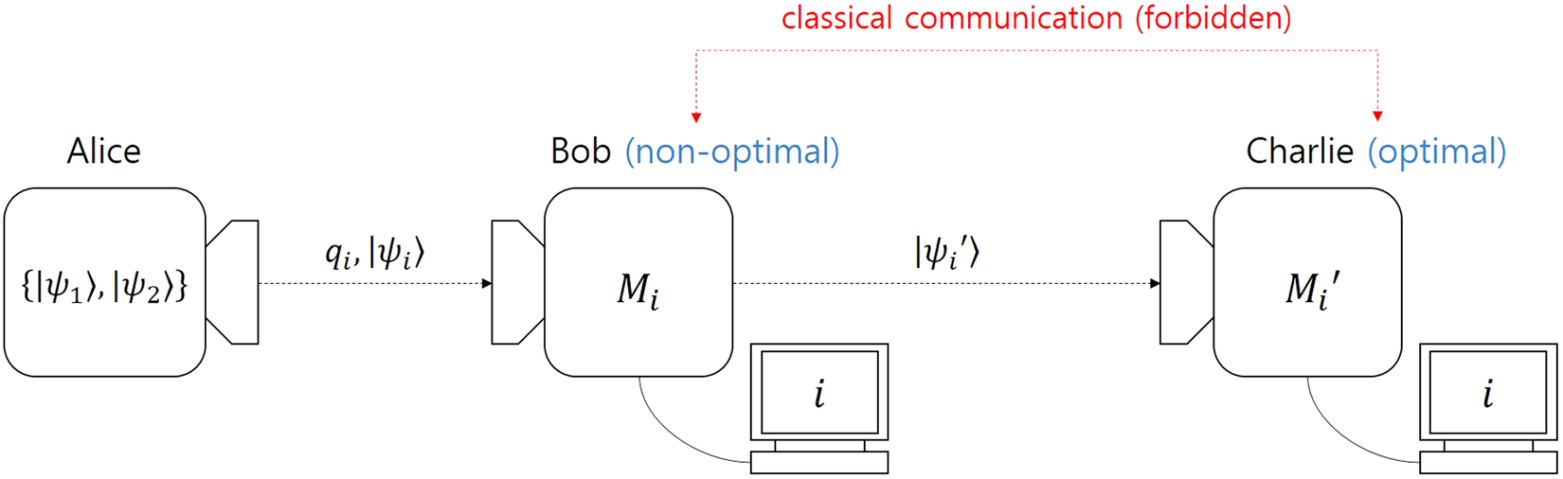
The scenario of sequential state discrimination, comprising a sender Alice and two receivers Bob and Charlie. Alice prepares a pure quantum state $|{\psi }_{i}\rangle$ with prior probabilities *q*_*i*_ and sends it to Bob. Bob performs non-optimal unambiguous discrimination on Alice’s pure state. Charlie does optimal unambiguous discrimination on Bob’s post-measurement state. Classical communication is not allowed between Bob and Charlie.

Let us briefly explain the case of two pure states in sequential state discrimination, as shown in Fig. 1. A sender Alice prepares a quantum state $|{\psi }_{i}\rangle$ out of non-orthogonal two pure states $\{|{\psi }_{1}\rangle ,|{\psi }_{2}\rangle \}$, with a prior probability *q*_*i*_. Two receivers Bob and Charlie should be able to discriminate the quantum states of Alice, without error. Therefore, they must perform unambiguous discrimination.

If Bob and Charlie can use a classical communication, the best strategy of Bob and Charlie is that without error, Bob optimaly discriminates Alice’s pure states and sends his measurement result to Charlie, through classical communication. Even though this strategy offers high success probability, the usage of classical communication is vulnerable to eavesdropping. Therefore, for security reasons, classical communication should not be allowed between Bob and Charlie. This condition is an important constraint on sequential state discrimination. In this process, a receiver-Bob, should use non-optimal unambiguous discrimination on Alice’s quantum state $|{\psi }_{i}\rangle$^28,30^. When Bob obtains a conclusive result, he sends his post-measurement state $|{\psi ^{\prime} }_{i}\rangle$ to another receiver Charlie. Charlie should then perform optimal unambiguous discrimination on the post-measurement state of Bob. When Bob and Charlie obtain a conclusive result, they can share the information encoded in Alice’s quantum state. The probability that Bob and Charlie obtain the conclusive result is given by

1 $${P}_{s}^{(B,C)}={q}_{1}\langle {\psi }_{1}|{\hat{M}}_{1}|{\psi }_{1}\rangle \,\langle {\psi ^{\prime} }_{1}|{\hat{M}^{\prime} }_{1}|{\psi ^{\prime} }_{1}\rangle +{q}_{2}\langle {\psi }_{2}|{\hat{M}}_{2}|{\psi }_{2}\rangle \,\langle {\psi ^{\prime} }_{2}|{\hat{M}^{\prime} }_{2}|{\psi ^{\prime} }_{2}\rangle .$$

where $\{{\hat{M}}_{0},{\hat{M}}_{1},{\hat{M}}_{2}\}$ ($\{{\hat{M}^{\prime} }_{0},{\hat{M}^{\prime} }_{1},{\hat{M}^{\prime} }_{2}\}$) denotes the POVM of Bob (Charlie) and ${\hat{M}}_{0}$ (${\hat{M}^{\prime} }_{0}$) describes the POVM element corresponding to the inconclusive result of Bob (Charlie). When $i\ne 0$, ${\hat{M}}_{i}$ (${\hat{M}^{\prime} }_{i}$) is the POVM element corresponding to a conclusive result *i* of Bob (Charlie).

As previously indicated, the purpose of sequential state discrimination is to maximize the success probability of Eq. (1). If the prior probabilities are identical and $s=|\langle {\psi }_{1}|{\psi }_{2}\rangle |\le 3-2\sqrt{2}$, the optimal success probability when Bob and Charlie discriminate every pure state of Alice is expressed by^26^

2 $${P}_{s}^{(B,C)\ast }={(1-\sqrt{s})}^{2}.$$

Meanwhile, if $s > 3-2\sqrt{2}$, the optimal success probability when Bob and Charlie discriminate only one of two pure states of Alice is given by^27^

3 $${P}_{s}^{(B,C)\ast }=\frac{1}{2}{(1-s)}^{2}.$$

If the two prior probabilities *q*_1_ and *q*_2_ are not equal, it is difficult to analytically determine the optimally success probabilty. However, When Bob and Charlie discriminate only one of the two pure states of Alice, the success probability is expressed by^30^

4 $${P}_{s}^{(B,C)\ast }=\,{\rm{\max }}\,\{{q}_{1},{q}_{2}\}{(1-s)}^{2}.$$

when the prior prabability is identical, Eq. (4) becomes Eq. (3). The detailed explanation can be found in the Method and the result of M. Namkung *et al*.^30^.

### Optical design of sequential state discrimination

In this section, we propose optical designs for sequential state discrimination of binary coherent states. Firstly, using the models of Banaszek^33^ and Huttner *et al*.^34^, we show how to implement a generalized measurement to perform unambiguous discrimination. We call the models the *Banaszek model* and the *Huttner*-*like model*. Based on the models, we demonstrate sequential state discrimination of two coherent states, which can be performed as a proof-of-principle experiment. In addition, since coherent states are robust to a noisy environment^36^, the optical designs for sequential state discrimination of two coherent states can be applied in the construction of a robust multiparty QKD. Specially, when the Huttner-like model is used, the security of the multiparty QKD can be improved.

#### Design idea

The discrimination of non-orthogonal coherent states is a subject of study which has generated significant research interest^43^. In the case of minimum error discrimination of coherent states, the way to obtain the Helstrom bound is known^1^. However, the an appropriate practical receiver for achieving the Helstrom bound has not been identified. In 1973, Dolinar suggested a receiver which can perform minimum error discrimination of two coherent states-using a beam combiner, photon detector, and feedback control^35^. Subsequently, many researchers attempted to implement receivers using a similar idea which can facilitate minimum error discrimination of *N*(>2) coherent states^36–38^. However, it has been shown that any receivers similar to Dolinar’s cannot achieve the Helstrom bound^39^. M. Sasaki and O. Hirota^44^ attempted to achieve minimum error discrimination of two coherent states by implementing rank-1 projective measurement. Meanwhile, since the unitary operator used in the result of M. Sasaki and O. Hirota^44^ was non-Gaussian, it could not be implemented using linear optics^45^. In order to achieve the Helstrom bound, M. P. da Silva *et al*.^40^ exploited an interaction between the quantum computer and the coherent states. However, it is difficult to implement the quantum computer in practice. Meanwhile, R. Han *et al*.^41^ used Jaynes-Cummings interaction^46^ between light and a two-level atom in order to demonstrate nearly minimum error discrimination of two coherent states with different phases.

K. Banaszek^33^ and B. Huttner *et al*.^34^ showed that a beam splitter, photon detector, and beam combiner can be used to perform optimal unambiguous discrimination of two coherent states with identical prior probability. In fact, even though unambiguous discrimination of coherent states has been studied less extensively than minimum error discrimination of coherent states^33,34,47^, it may be implemented easier than minimum error discrimination of coherent states. For example, Doliner’s receiver is very difficult to implement^45^ due to electric feedback, but optimal unambiguous discrimination can be implemented by a linear optics - even when the priror probability is not identical, without considering electric feedback, a quantum computer^40^ or Jaynes-Cummings interaction^41^.

Therefore, using the fact that optimal unambiguous discrimination of two coherent states with an arbitrary prior probability can be accomplished using a beam splitter, beam combiner, and photon detector, we will show that the post-measurement state of the non-orthogonal two coherent states can be obtained by utilizing the method of K. Banaszek^33^ and B. Huttner *et al*.^34^. The post-measurement state is needed for sequential state discrimination of Bob and Charlie.

**Figure 2:**
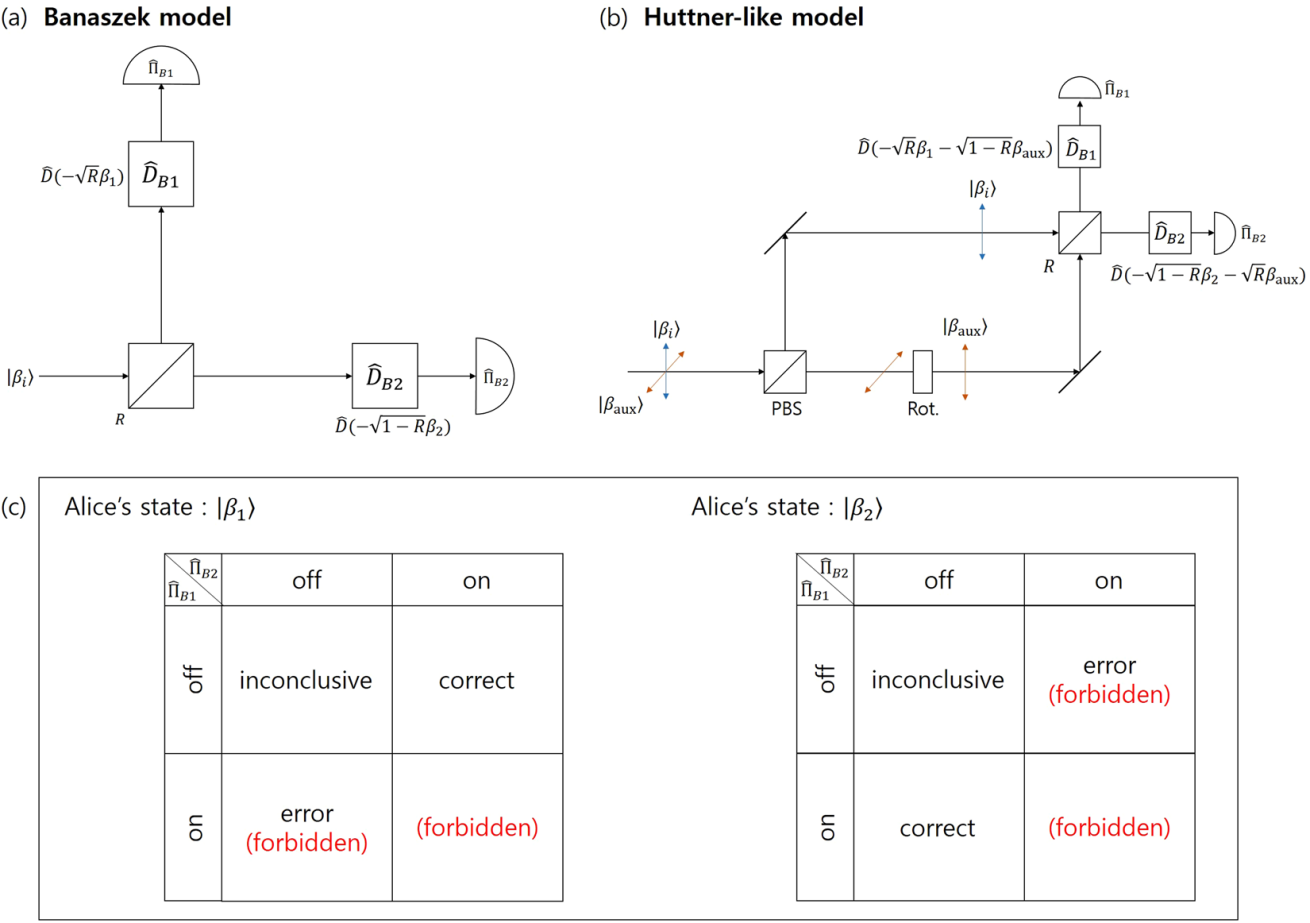
The structure of the Banaszek model and the Huttner-like model. (**a**) The Banaszek model. (**b**) The Huttner-like model. Both models use beam splitters, beam combiners (displacement operator), and photon detectors. (**c**) The measurement result in both models, in terms of on/off of photon detector.

*Implementing optimal unambiguous discrimination*. First, let us propose the optical model of Fig. 2(a) by modifying the result of K. Banaszek^33^. The model will be called Banaszek model. It is composed of a beam splitter with a reflection ratio $R\in [0,1]$, a beam combiner, and a photon detector. The beam combiner can be described by a displacement operator $\hat{D}(\gamma )={e}^{\alpha {\hat{a}}^{\dagger }-{\alpha }^{\ast }\hat{a}}$ which satisfies $\hat{D}(\gamma )|\beta \rangle ={e}^{(\gamma {\beta }^{\ast }-{\gamma }^{\ast }\beta )/2}|\gamma +\beta \rangle$ (Here, we omits the phase term, for convenience). And $\hat{a}({\hat{a}}^{\dagger })$ is an annihilation operator (creation operator). The displacement operator $\hat{D}(\gamma )$ can be implemented by 50/50 beam splitter and laser source^45^: In order to explain how to work in a detailed way, let us denote two input ports of 50/50 beam splitter as *a* and *b*. First, coherent state $|\alpha \rangle$ is sent to input port *a*. Next, coherent state $|\gamma ^{\prime} \rangle$, having amplitude of $\gamma ^{\prime} =(\sqrt{2}-1)\alpha +\sqrt{2}\gamma$, is sent to input port *b*. Then, 50/50 beam splitter transforms input coherent state ${|\alpha \rangle }_{a}\otimes {|\gamma ^{\prime} \rangle }_{b}$ into output coherent state ${|\alpha +\gamma \rangle }_{a}\otimes {|(\sqrt{2}-1)\alpha -\gamma \rangle }_{b}$. Finally, coherent state $|\alpha +\gamma \rangle$ of output port *a* is chosen. Alice prepares a coherent state $|{\beta }_{i}\rangle \in \{|{\beta }_{1}\rangle ,|{\beta }_{2}\rangle \}$ with a prior probability *q*_*i*_, where ${\beta }_{i}\in {\mathbb{C}}$. After Alice’s coherent state $|{\beta }_{i}\rangle$ goes through beam splitter, the coherent state becomes $|\sqrt{R}{\beta }_{i}\rangle \otimes |\sqrt{1-R}{\beta }_{i}\rangle$. The partial coherent state reflected by the beam splitter goes through the displacement operator ${\hat{D}}_{{\rm{B}}1}=\hat{D}(\,-\,\sqrt{R}{\beta }_{1})$. Meanwhile, the partial coherent state is transmitted through the beam splitter and goes through the displacement operator ${\hat{D}}_{{\rm{B}}2}=\hat{D}(\,-\,\sqrt{1-R}{\beta }_{2})$. When $i=1$, two photon detectors measure $|0\rangle \otimes |\sqrt{1-R}({\beta }_{1}-{\beta }_{2})\rangle$. The photon detector used in the Banaszek model determines whether the number of photons is zero or not. That is, the *i* (∈ {1, 2})-th photon detector is described by projective measurement $\{{\hat{{\rm{\Pi }}}}_{{\rm{B}}i}^{({\rm{off}})},{\hat{{\rm{\Pi }}}}_{{\rm{B}}i}^{({\rm{on}})}\}$. Here, measurement element becomes ${\hat{{\rm{\Pi }}}}_{{\rm{B}}i}^{(\mathrm{off})}=|0\rangle \langle 0|,\,{\hat{{\rm{\Pi }}}}_{{\rm{B}}i}^{(\mathrm{on})}={\mathbb{I}}-|0\rangle \langle 0|$. If the measurement result of the two photon detectors is (off, on), Bob can detect that the coherent state prepared by Alice is $|{\beta }_{1}\rangle$. When $i=2$, two photon detectors measure $|-\sqrt{R}({\beta }_{1}-{\beta }_{2})\rangle \otimes |0\rangle$. If the measurement result is (on, off), Bob can determine that the coherent state prepared by Alice is $|{\beta }_{2}\rangle$. When for arbitrary *i*, the measurement result of the two photon detectors is (off, off), Bob cannot obtain information on Alice’s coherent state. In other word, the measurement result of (off, off) corresponds to the inconclusive result. In the case of the ideal Banaszek model, if Alice’s coherent state is $|{\beta }_{1}\rangle$ ($|{\beta }_{2}\rangle$), the measurement result of Bob cannot be (on, off)((off, on)). Therefore, the Banaszek model can discriminate the two coherent states without error and the measurement result of the two photon detectors cannot be (on, on) regardless of the coherent state. The measurement result of the photon detector is summarized in the table of Fig. 2(c). The average failure probability of Bob is given as follows:

5 $$\begin{array}{rcl}{P}_{f}^{(B)} & = & {q}_{1}{\rm{Tr}}[|0\rangle \langle 0|\otimes |\sqrt{1-R}({\beta }_{1}-{\beta }_{2})\rangle \langle \sqrt{1-R}({\beta }_{1}-{\beta }_{2})|{\hat{{\rm{\Pi }}}}_{{\rm{B}}1}^{({\rm{off}})}\otimes {\hat{{\rm{\Pi }}}}_{{\rm{B}}2}^{({\rm{off}})}]\\  &  & +\,{q}_{2}{\rm{Tr}}[|\,-\,\sqrt{R}({\beta }_{1}-{\beta }_{2})\rangle \langle \,-\,\sqrt{R}({\beta }_{1}-{\beta }_{2})|\otimes |0\rangle \langle 0|{\hat{{\rm{\Pi }}}}_{{\rm{B}}1}^{({\rm{off}})}\otimes {\hat{{\rm{\Pi }}}}_{{\rm{B}}2}^{({\rm{off}})}]\\  & = & {q}_{1}{e}^{-(1-R)|{\beta }_{1}-{\beta }_{2}{|}^{2}}+{q}_{2}{e}^{-R|{\beta }_{1}-{\beta }_{2}{|}^{2}}.\end{array}$$

The condition that allows the maximum of Eq. (5) to be determined can be found using $\partial {P}_{f}^{(B,C)}/\partial R=0$:

6 $${R}^{\ast }=\frac{1}{2}+\frac{1}{|{\beta }_{1}-{\beta }_{2}{|}^{2}}\,\mathrm{ln}\,\sqrt{\frac{{q}_{2}}{{q}_{1}}}.$$

when $0 < {R}^{\ast } < 1$, Bob can discriminate two coherent states of Alice. In this case, the minimum failure probability of Bob is given by

7 $${P}_{f}^{(B)\ast }=2\sqrt{{q}_{1}{q}_{2}}{e}^{-|{\beta }_{1}-{\beta }_{2}{|}^{2}/2},\,0 < {R}^{\ast } < 1.$$

If ${R}^{\ast }=0$, Bob discriminates only $|{\beta }_{1}\rangle$. In this case, the minimum failure probability of Bob is given by

8 $${P}_{f}^{(B)\ast }={q}_{1}{e}^{-|{\beta }_{1}-{\beta }_{2}{|}^{2}}+{q}_{2},\,{R}^{\ast }=0.$$

Meanwhile, when ${R}^{\ast }=1$, Bob discriminates only $|{\beta }_{2}\rangle$. In this case, the minimum failure probability of Bob is

9 $${P}_{f}^{(B)\ast }={q}_{1}+{q}_{2}{e}^{-|{\beta }_{1}-{\beta }_{2}{|}^{2}},\,{R}^{\ast }=1.$$

here, ${e}^{-|{\beta }_{1}-{\beta }_{2}{|}^{2}/2}=|\langle {\beta }_{1}|{\beta }_{2}\rangle |$. Equations (7–9) is the analytic minimum failure probability obtained by G. Jaeger and A. Shimony^11^.

We will now examine the details of the receiver proposed by Huttner *et al*.^34^. In this receiver, Alice encodes her message into orthogonally polarized cohrent signals $\{|\alpha \rangle ,|\,-\,\alpha \rangle \}$. The coherent state is combined with horizontally polarized auxilliary coherent light $|\,-\,\alpha \rangle$ and is sent to Bob. The coherent signal is reflected off in Bob’s polarized beam splitter. Meanwhile, the auxilliary coherent light is transmitted through the polarized beam splitter. The polarization of the auxilliary coherent state which passes through the polarized beam splitter is changed to a vertical polarization when it traverses through the rotator.

When the coherent signal prepared by Alice is $|\alpha \rangle$, a photon is not detected in the first photon detector. Meanwhile, if Alice prepares $|\,-\,\alpha \rangle$, a photon is not detected in the second photon detector. Therefore, similar to the Banaszek model, this model can discriminate coherent signals of Alice without eror, according to the result of the measurement in two photon detectors of Bob, which is (off, on) or (on, off) (See Fig. 2(c)). If Eve wants to eavesdrop on Alice’s message, the scenario can be described as follows: Firstly, Eve decouples the coherent signal and auxilliary coherent light using a polarized beam splitter. Then, setting up the beam splitter in the part of coherent signal, Eve may obtain a part of Alice’s coherent signal. If Eve can obtain information about Alice’s coherent signal, the amplitude of the coherent signal and the auxilliary coherent light cannot be the same. Therefore, Alice and Bob can detect the existence of eavesdropper Eve.

However, when a prior probability of two coherent signals is arbitrary, Bob should use an appropriate beam combiner in front of the two photon detectors, in order to perform unambiguous discrimination. The modified model is shown in Fig. 2(b), and is called the Huttner-like model. In Fig. 2(b), Alice combines one of the non-orthogonal two polarized coherent states $\{|{\beta }_{1}\rangle ,|{\beta }_{2}\rangle \}$ with the horizontally polarized coherent state $|{\beta }_{{\rm{aux}}}\rangle$ and sends it to Bob. Here, *q*_*i*_ is the prior probability of the coherernt state $|{\beta }_{i}\rangle$. In fact, coherent signal is reflected at polarized beam splitter. Meanwhile, auxiliary coherent light penetrates polarized beam splitter. The polarization of the auxillary coherent light passing through the polarized beam splitter is changed into a vertical polarization by the rotator. Therefore, coherent signal and auxiliary coherent light can interact with each other at the beam splitter. Bob’s beam splitter produces the coherent state $|\sqrt{R}{\beta }_{i}+\sqrt{1-R}{\beta }_{{\rm{aux}}}\rangle \otimes |\sqrt{1-R}{\beta }_{i}-\sqrt{R}{\beta }_{{\rm{aux}}}\rangle$ at two output ports. The coherent state is transmitted through Bob’s displacement operator ${\hat{D}}_{{\rm{B}}1}=\hat{D}(\,-\,\sqrt{R}{\beta }_{1}-\sqrt{1-R}{\beta }_{{\rm{aux}}})$ and ${\hat{D}}_{{\rm{B}}2}=\hat{D}(\,-\,\sqrt{1-R}{\beta }_{2}-\sqrt{R}{\beta }_{{\rm{aux}}})$. Eventually, Bob’s on/off detectors locally measure coherent state $|\sqrt{R}({\beta }_{i}-{\beta }_{1})\rangle \otimes |\sqrt{1-R}({\beta }_{i}-{\beta }_{2})\rangle$. When Alice’s coherent signal is $|{\beta }_{1}\rangle$, a photon is not detected in the first photon detector. If Alice’s coherent signal is $|{\beta }_{2}\rangle$, a photon is not detected in the second photon detector. Therefore, without error, Bob can discriminate Alice’s coherent signals by the result of measurements based on the (off, on) or (on, off) of the detector. In the Huttner-like model, the average failure probability is the same as Eq. (5). Smilar to the Banaszek model, the minimum failure probability coincides with the analytic limit^11^.

**Figure 3:**
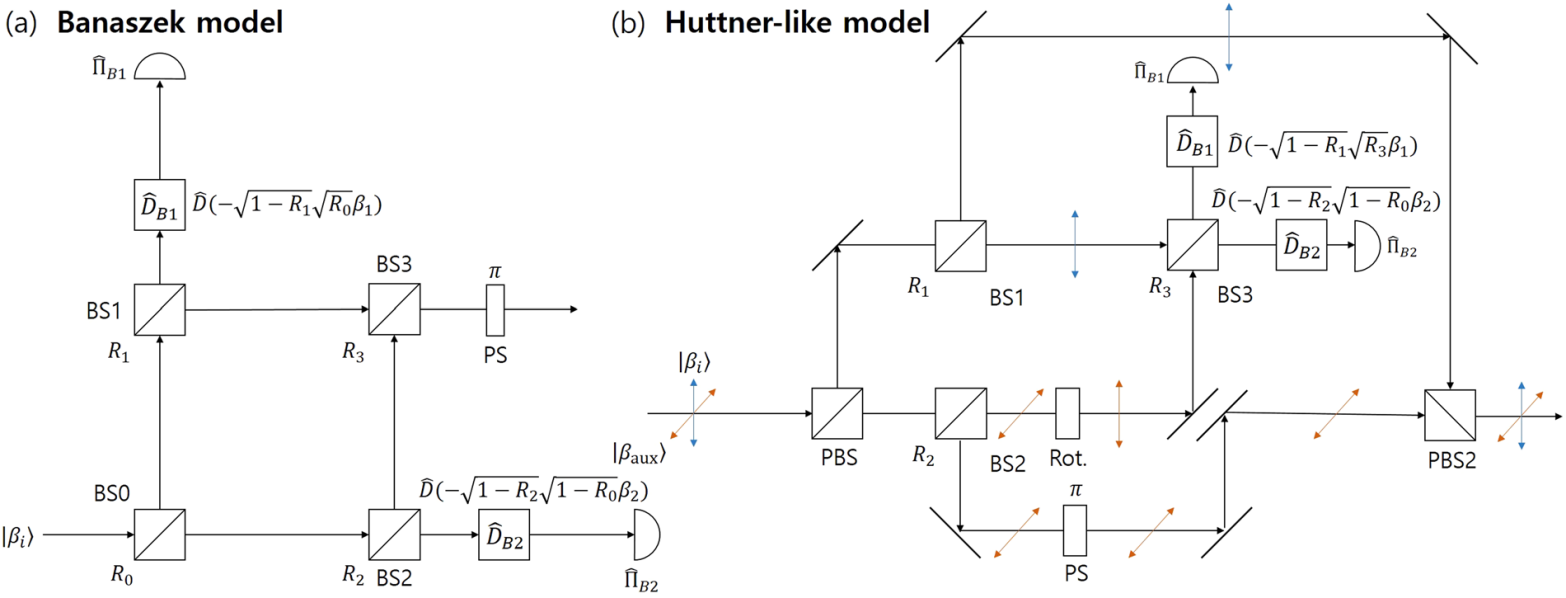
(**a**) Banaszek model and (**b**) Huttner-like model, which can build post-measurement state.

*Constructing post*-*measurement states*. In this section, the method to produce a post-measurement state in the Banaszek model and Huttner-like model will be explained. In the Banaszek model which is shown in Fig. 3(a), two kinds of post-measurement state can be obtained by adding three beam splitters (BS1, BS2, BS3). In Fig. 3(a), a part of the partial coherent state is reflected on BS1 and BS2. These reflected components which meet at BS3, represent a post-measurement state. In order for light to be confined to only one port out of the two output ports of BS3, the reflection ratio *R*_3_ is determined as follows:

10 $${R}_{3}=\frac{{R}_{2}(1-{R}_{0})}{{R}_{1}{R}_{0}+{R}_{2}(1-{R}_{0})}.$$

**Figure 4:**
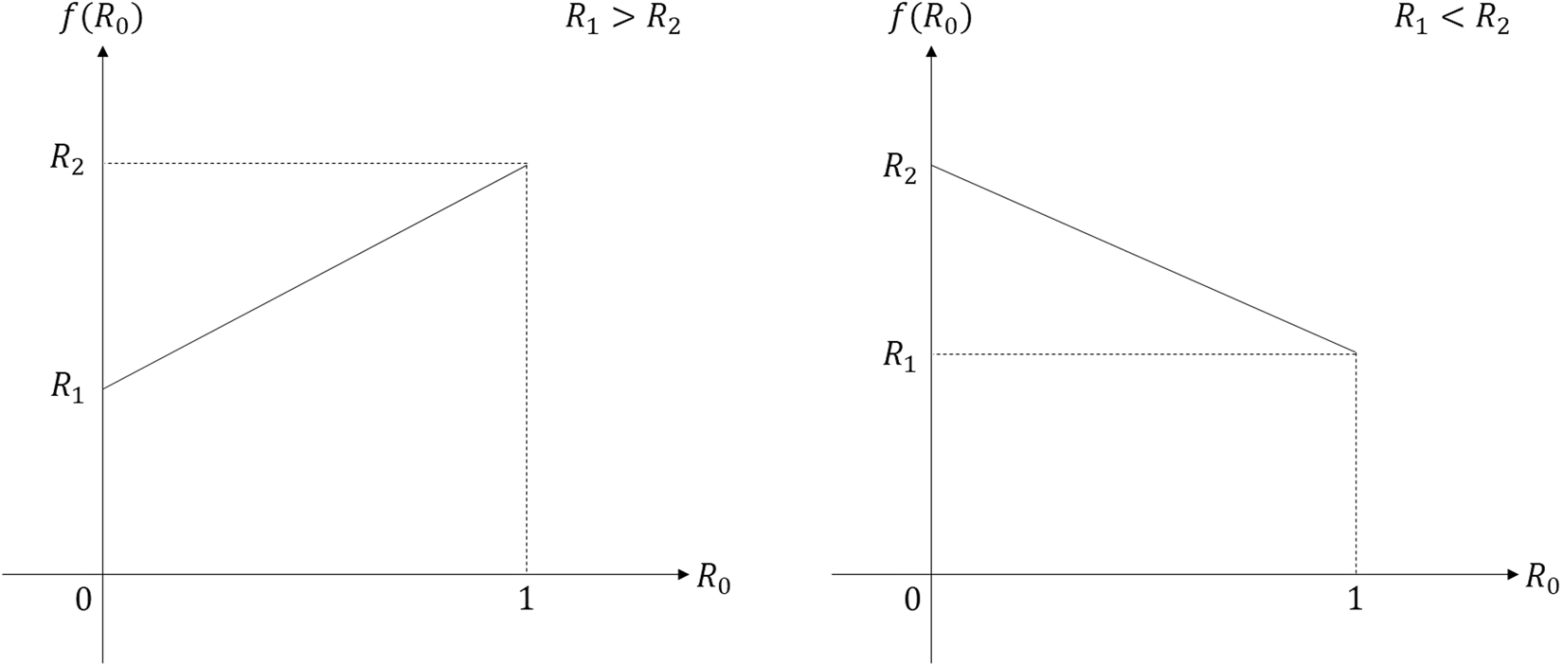
The value of *f*(*R*_0_), in terms of *R*_1_ and *R*_2_.

The light which comes from BS3 goes through a *π* phase shifter. When Alice prepares a coherent state $|{\beta }_{i}\rangle$, Bob’s Banaszek model produces a post-measurement state $|\sqrt{f}{\beta }_{i}\rangle$, which is sent to Charlie. Here $f={R}_{1}{R}_{0}+{R}_{2}(1-{R}_{0})$. When ${R}_{1} > {R}_{2}$, *f* satisfies the inequality ${R}_{2}\le f\le {R}_{1}$. Meanwhile, when ${R}_{1} < {R}_{2}$, we have ${R}_{1}\le f\le {R}_{2}$ (See Fig. 4). By combining the two inequalities, we can obtain the following inequality:

11 $${\rm{\min }}\,\{{R}_{1},{R}_{2}\}\le f\le \,{\rm{\max }}\,\{{R}_{1},{R}_{2}\},\,\forall \,{R}_{1},{R}_{2}\in [0,1].$$

If ${R}_{1}={R}_{2}$, by Eq. (11) we can have an equality of *f*. Since when ${R}_{1}={R}_{2}=0$, we have $f=0$, the overlap of two post-measurement states $|\sqrt{f}{\beta }_{1}\rangle$ and $|\sqrt{f}{\beta }_{2}\rangle$ becomes one. This implies that the post-measurement states of Bob cannot be discriminated, because Bob optimally discriminates Alice’s two coherent states. Meanwhile, when ${R}_{1}={R}_{2}=1$, we have $f=1$, which means that the overlap of the post-measurement states is identical to that of the two coherent states of Alice, since Bob did not obtain any information about Alice’s coherent states. In fact, by Eq. (11), BS1, BS2, and BS3 of the Banaszek model can control the overlap of the post-measurement states, which lies in $|\langle {\beta }_{1}|{\beta }_{2}\rangle |\le |\langle \sqrt{f}{\beta }_{1}|\sqrt{f}{\beta }_{2}\rangle |\le 1$. This argument is consistent with the result of J. A. Bergou *et al*.^26^.

The Huttner-like model, which can build a post-measurement state is more complex than the Banaszek model. This is because, unlike the Banaszek model, the Huttner-like model uses two different polarized coherent light. In the latter, which is shown in Fig. 3(b), the beam splitters BS1 and BS2 are installed for a coherent signal and auxilliary coherent light. The coherent light passing through BS2 goes to the rotator (Rot). Therefore, the lights that traverses through BS1 and BS2 meet at BS3. Meanwhile, the reflected light at BS1 and BS2 meet at PBS2. In the process, the reflected light at BS2 traverses the phase shifter (PS). The two optical cmponents which meet at PBS2, construct a post-measurement state of Bob.

When ${R}_{1}={R}_{2}=0$, Bob’s post-measurement state becomes a vacuum state. However, if ${R}_{1}={R}_{2}=1$, Bob does not perform unambiguous discrimination. Therefore, the reflection ratios of BS1 and BS2 determine the trade-off between the ability of Bob to perform unambiguous discrimination and the overlap of post-measurement states.

#### Implementing optimal sequential state discrimination

In the previous section, we described the method to discriminate two coherent states without error, by using the Banaszek model or the Huttner-like model. In addition, the post-measurement state of non-orthogonal coherent state can be produced using those two models. We can then construct an optimal sequential state discrimination of two coherent states with arbitrary prior probability, by applying those models.

**Figure 5:**
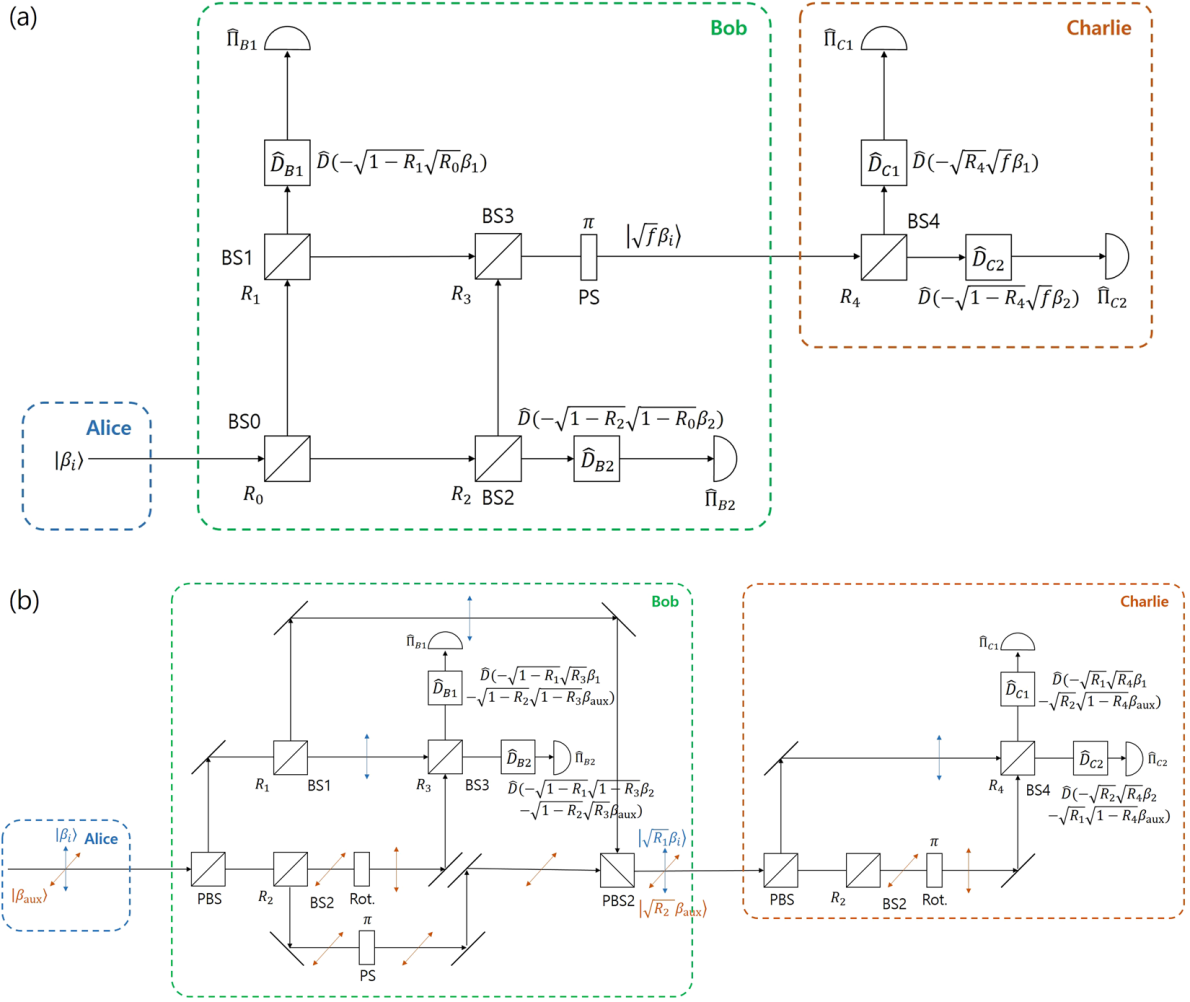
Sequential state discrimination using (**a**) Banaszek model and (**b**) Huttner-like model.

Herein, we propose the optical design for sequential state discrimination of two coherent states, based on the Banaszek model or the Huttner-like model, which can be seen in Fig. 5. The optical design is one of the *main results*. In Fig. 5(a), Bob constructs the Banaszek model to perform non-optimal unambiguous discrimination then sends a post-measurement state $\{|\sqrt{f}{\beta }_{1}\rangle ,|\sqrt{f}{\beta }_{2}\rangle \}$ to Charlie. Charlie builds the Banaszek model to perform optimal unambiguous discrimination on Bob’s post-measurement state. The success probability ${P}_{s}^{(B,C)}$ that Bob and Charlie successfully perform sequential state discrimination depends on the reflection ratio ${R}_{0},{R}_{1},{R}_{2}$, and ${R}_{4}$ of the beam splitter BS0, BS1, BS2, and BS4 respectively in the Banaszek model of Bob and Charlie. The success probability ${P}_{s}^{(B,C)}$ can be expressed by ${P}_{s}^{(B,C)\ast }={{\rm{\max }}}_{\{{R}_{0},{R}_{1},{R}_{2},{R}_{4}\}}\,{P}_{s}^{(B,C)}$. However, since ${P}_{s}^{(B,C)}$ is a function of four variables ${R}_{0},{R}_{1},{R}_{2}$, and ${R}_{4}$, and is difficult to obtain analytically. In this article, we use constrained optimization^48^. Here, the constraints are inequalities where every reflection ratio exists in the region of $[0,1]$.

Figure 5(b) shows the Huttner-like model constructed by Bob and Charlie. The Huttner-like model of Bob discriminates non-optimally the two coherent states of Alice without error. However, the Huttner-like model of Charlie discriminates optimally the post-measurement states of Bob without error. The optimal success probability of the Huttner-like model depends on the reflection ratios of ${R}_{1},{R}_{3}$, and ${R}_{4}$ in beam splitters BS1, BS3, and BS4 of Bob and Charlie, which is given by ${P}_{s}^{(B,C)\ast }={{\rm{\max }}}_{\{{R}_{1},{R}_{3},{R}_{4}\}}\,{P}_{s}^{(B,C)}$.

##### **Example 1**.

(**Binary phase-key-shifting** (**BPSK**) **coherent states**) In this example, we consider sequential state discrimination of BPSK signals. The *M*–ary PSK coherent state is defined as:

12 $$|{\alpha }_{m}\rangle =|\alpha {u}^{m}\rangle ,\,u=\exp (\frac{2\pi i}{M}).$$

here, $\alpha \in {\mathbb{R}}$. The *M*–ary PSK coherent state is linearly independent and has a symmetry^1,45^, expressed as:

13 $$|{\alpha }_{m}\rangle =\hat{V}|{\alpha }_{0}\rangle ,\,\hat{V}=\exp (\frac{2\pi i}{M}\hat{n}).$$

here, $\hat{n}$ is the photon number operator. In the sequential discrimination of the BPSK signal ($M=2$), Alice prepares one of the BPSK coherent states $\{|\,+\,\alpha \rangle ,|\,-\,\alpha \rangle \}$, with an arbitrary prior probability *q*_+_ (*q*_−_). That is, ${q}_{+}\ne {q}_{-}$. When Bob and Charlie construct the Banaszek model, the success probability of sequential state discrimination is given as:

14 $${P}_{s}^{(B,C)}={q}_{+}\{1-{e}^{-4(1-{R}_{2})(1-{R}_{0}){\alpha }^{2}}\}\{1-{e}^{-4(1-{R}_{4})f{\alpha }^{2}}\}+{q}_{-}\{1-{e}^{-4(1-{R}_{1}){R}_{0}{\alpha }^{2}}\}\{1-{e}^{-4{R}_{4}f{\alpha }^{2}}\}.$$

Meanwhile, when Bob and Charlie construct the Huttner-like model, the success probability for sequential state discrimination is given by

15 $${P}_{s}^{(B,C)}={q}_{+}\{1-{e}^{-4(1-{R}_{3})(1-{R}_{1}){\alpha }^{2}}\}\{1-{e}^{-4(1-{R}_{4}){R}_{1}{\alpha }^{2}}\}+{q}_{-}\{1-{e}^{-4{R}_{3}(1-{R}_{1}){\alpha }^{2}}\}\{1-{e}^{-4{R}_{4}{R}_{1}{\alpha }^{2}}\}.$$

**Figure 6:**
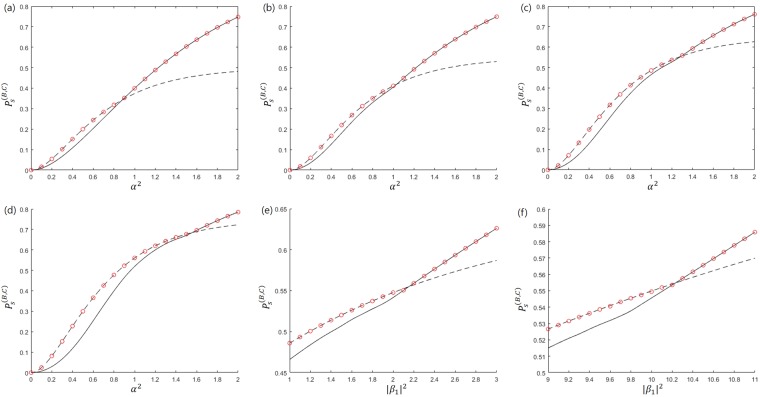
The optimal success probability of two pure states and the optimal success probability from the Banaszek model and the Huttner-like model. The black solid line (black dashed line) denotes the optimal success probability when Bob and Charlie discriminate every pure states of Alice (only one of two pure states of Alice). The red circle denotes the optimal success probability of optical models. (**a**–**d**) Denotes the case of BPSK signal when (**a**) ${q}_{+}=0.5$, (**b**) ${q}_{+}=0.55$, (**c**) ${q}_{+}=0.6$, (**d**) ${q}_{+}=0.65$. (**e**,**f**) Denote the case of coherent states when (**e**) $|{\beta }_{2}|=\sqrt{3}$, ${\rm{\Delta }}=-\,\pi /2$ and (**f**) $|{\beta }_{2}|=\sqrt{6}$, ${\rm{\Delta }}=\pi /4$, with ${q}_{+}=0.65$.

Here, the auxiliary coherent light is assumed to be $|-\alpha \rangle$^34^. If two prior probabilities *q*_+_ and *q*_−_ are identical, one can find an analytic condition reaching to the result of J. A. Bergou *et al*.^26^ in both the Banaszek model and the Huttner-like model. (The detailed analysis is given in the Method section). When the prior probabilities are different, the optimal success probability is shown in Fig. 6. In this figure, the solid line shows the optimal success probability when Bob and Charlie discriminate the every coherent state of Alice. The solid line in the case of ${q}_{+}={q}_{-}$ coincides with the result of J. A. Bergou *et al*.^26^:

16 $${P}_{s}^{(B,C)\ast }={\{1-\sqrt{\langle -\alpha |\alpha \rangle }\}}^{2}={(1-{e}^{-{\alpha }^{2}})}^{2}.$$

The dashed line shows the optimal success probability when Bob and Charlie discriminate only one of two coherent states of Alice. In that case, the optimal success probability for different prior probabilities is expressed by^27,28,30^

17 $${P}_{s}^{(B,C)\ast }=\frac{1}{2}{\{1-\langle -\alpha |\alpha \rangle \}}^{2}=\frac{1}{2}{(1-{e}^{-2{\alpha }^{2}})}^{2}.$$

In Fig. 6, the red circle shows the optimal success probability when Bob and Charlie construct the Banaszek model (Eq. (14)) and the Huttner-like model (Eq. (15)). From Fig. 6(a–d), we can see that optimal sequential state discrimination can be achieved using the Banaszek model or the Huttner-like model. This result implies that the proof-of-principle experiment of sequential state discrimination can be performed using not only polarized photon states^32^ but also coherent states. Therefore, we can obtain multiparty QKD of coherent states by sequential state discrimination of these states. Since every receiver should discriminate every quantum state of the sender^26^ in the case of QKD, the region of large *α* is more useful for multiparty QKD. This is because when *α* is large, the solid line is greater than the dashed line. However, as the difference between prior probabilities becomes large, the minimum of *α* increases when the solid line is greater than or equal to the dashed line. However, in the case of equal prior probability, coherent states of a small amplitude can be used for multiparty QKD.

##### **Example 2**.

(**Arbitrary two coherent states**) Let us consider the sequential state discrimination of two arbitrary coherent states $\{|{\beta }_{1}\rangle ,|{\beta }_{2}\rangle \}$ when ${\beta }_{1}\in {\mathbb{C}}$ and ${\beta }_{2}\in {\mathbb{C}}$ are different. Since ${\beta }_{i}=|{\beta }_{i}|\,\cos \,{\theta }_{i}+i|{\beta }_{i}|\,\sin \,{\theta }_{i}$, $|{\beta }_{1}-{\beta }_{2}{|}^{2}$ can be expressed as:

18 $$|{\beta }_{1}-{\beta }_{2}{|}^{2}=|{\beta }_{1}{|}^{2}+|{\beta }_{2}{|}^{2}-2|{\beta }_{1}|\,|{\beta }_{2}|\,\cos \,{\rm{\Delta }}\theta ,\,{\rm{\Delta }}\theta ={\theta }_{1}-{\theta }_{2}.$$

Firstly, we consider the two cases of $|{\beta }_{2}|=\sqrt{3},\,{\rm{\Delta }}\theta =-\,\pi /2$ (Fig. 6(e)) and $|{\beta }_{2}|=\sqrt{6},\,{\rm{\Delta }}\theta =\pi /4$ (Fig. 6(f)). In Fig. 6, we assume ${q}_{1}=0.65$ and ${q}_{2}=0.35$. When Bob and Charlie construct the Banaszek model, the success probability is given by:

19 $$\begin{array}{rcl}{P}_{s}^{(B,C)} & = & {q}_{1}\{1-{e}^{(1-{R}_{2})(1-{R}_{0})|{\beta }_{1}-{\beta }_{2}{|}^{2}}\}\{1-{e}^{-(1-{R}_{4})f|{\beta }_{1}-{\beta }_{2}{|}^{2}}\}\\  &  & +\,{q}_{2}\{1-{e}^{-(1-{R}_{1}){R}_{0}|{\beta }_{1}-{\beta }_{2}{|}^{2}}\}\{1-{e}^{-{R}_{4}f|{\beta }_{1}-{\beta }_{2}{|}^{2}}\}.\end{array}$$

If Bob and Charlie use the Huttner-like model, the success probability is obtained by:

20 $$\begin{array}{rcl}{P}_{s}^{(B,C)} & = & {q}_{1}\{1-{e}^{-(1-{R}_{3})(1-{R}_{1})|{\beta }_{1}-{\beta }_{2}{|}^{2}}\}\{1-{e}^{-(1-{R}_{4}){R}_{1}|{\beta }_{1}-{\beta }_{2}{|}^{2}}\}\\  &  & +\,{q}_{2}\{1-{e}^{-{R}_{3}(1-{R}_{1})|{\beta }_{1}-{\beta }_{2}{|}^{2}}\}\{1-{e}^{-{R}_{4}{R}_{1}|{\beta }_{1}-{\beta }_{2}{|}^{2}}\}.\end{array}$$

In Fig. 6, we can see that both the Banaszek model and the Huttner-like model can achieve optimal success probability. The solid line (dashed line) shows the optimal success probability when Bob and Charlie discriminate every (only single) coherent state of Alice. The red circle displays the optimal success probability when Bob and Charlie construct the Banaszek model and the Huttner-like model. Also, Fig. 6 shows that in the case of asymmetric two coherent states, an extremely large amplitude is needed for multiparty QKD. This implies that two arbitrary coherent states is less useful for sequential state discrimination than BPSK signals.

#### Considering imperfect condition

Up to now, we have explained that sequential state discrimination can be optimally accomplished using the Banaszek model or the Huttner-like model. However, the quantum system may interact with the environment. For example, there can be a photon loss in coherent states^49^. The time evolution of the quantum state, which experiences a photon loss, is described by the following master equation^49^:

21 $$\frac{\partial \hat{\rho }}{\partial t}={\hat{ {\mathcal L} }}_{1}(\hat{\rho })+{\hat{ {\mathcal L} }}_{2}(\hat{\rho }).$$

here, ${\hat{ {\mathcal L} }}_{i}$ is the superoperator given by ${\hat{ {\mathcal L} }}_{1}(\hat{\rho })=\gamma \,{\sum }_{i}\,{\hat{a}}_{i}\hat{\rho }{\hat{a}}_{i}^{\dagger }$ and ${\hat{ {\mathcal L} }}_{2}(\hat{\rho })=-\,(\gamma /2)\,{\sum }_{i}\,({\hat{a}}_{i}^{\dagger }{\hat{a}}_{i}\hat{\rho }+\hat{\rho }{\hat{a}}_{i}^{\dagger }{\hat{a}}_{i})$. The real number *γ* denotes a decay rate. The time-evolution of the coherent state, described by Eq. (21), is the same as the case where a light goes through beam splitter with a transmition rate $\sqrt{\eta }={e}^{-\gamma t}$^50^.

**Figure 7:**
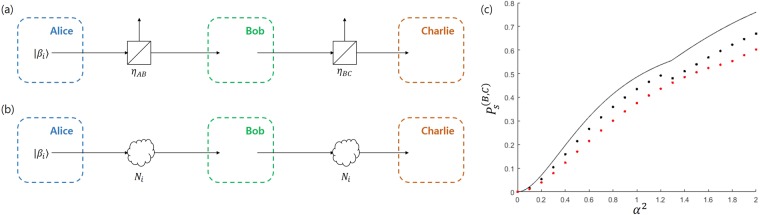
The scenario of sequential state discrimination in a realistic situation. (**a**) The case of the Banaszek model, where photon loss is represented by a beam splitter. (**b**) The case of the Huttner-like model. The model ${{\mathscr{N}}}_{i}$ of equivalent quantum channels is described in Fig. 8. (**c**) Denotes the success probability when the BPSK signal is considered. The black solid line is the optimal success probability in the ideal case. The black dotted line (red dotted line) denotes the success probability of the Banaszek model with uncorrected (corrected) displacement operator, when ${q}_{+}=0.65$, ${q}_{=}=0.35$.

*Imperfect Banaszek model*. Suppose that Alice prepares one of two BPSK coherent states $\{|\,+\,\alpha \rangle ,|\,-\,\alpha \rangle \}$ and sends it to Bob. If there is a photon loss in the quantum channel between Alice and Bob, the coherent state of Alice is given by

22 $$|\,\pm \,\alpha \rangle \to |\,\pm \,\sqrt{{\eta }_{AB}}\alpha \rangle .$$

here, $\sqrt{{\eta }_{AB}}\in [0,1]$ is the efficiency of the quantum channel between Alice and Bob. If the displacement operator is not modified to deal with a noise, there is an error in Bob’s on/off detector. Under the incomplete quantum channel (See Fig. 7), the coherent state preserves purity. When the displacement operator is corrected, quantum state discrimination can be performed without error. In the Banaszek model, the error can be eliminated when the displacement operator of Bob is modified as follows:

23 $$\begin{array}{rcl}{\hat{D}}_{B1}\to {\hat{D}^{\prime} }_{B1} & = & \hat{D}(\,-\,\sqrt{(1-{R}_{1}){R}_{0}}\alpha \sqrt{{\eta }_{AB}}),\\ {\hat{D}}_{B2}\to {\hat{D}^{\prime} }_{B2} & = & \hat{D}(\sqrt{(1-{R}_{2})(1-{R}_{0})}\alpha \sqrt{{\eta }_{AB}}).\end{array}$$

Bob sends the post-measurement state $|\,\pm \,\sqrt{f}\alpha \sqrt{{\eta }_{AB}}\rangle$ to Charlie. When there is a photon loss in the quantum channel between Bob and Charlie, the coherent state of Bob is obtained by:

24 $$|\,\pm \,\sqrt{f}\alpha \sqrt{{\eta }_{AB}}\rangle \to |\,\pm \,\sqrt{f}\alpha \sqrt{{\eta }_{AB}{\eta }_{BC}}\rangle .$$

here, $\sqrt{{\eta }_{BC}}\in [0,1]$ denotes the efficiency of quantum channel between Bob and Charlie. In the case, without error, Charlie can perform quantum state discrimination, by correcting displacement operator as follows:

25 $$\begin{array}{rcl}{\hat{D}}_{C1}\to {\hat{D}^{\prime} }_{C1} & = & \hat{D}(\,-\,\sqrt{{R}_{4}}\sqrt{f}\alpha \sqrt{{\eta }_{AB}{\eta }_{BC}}),\\ {\hat{D}}_{C2}\to {\hat{D}^{\prime} }_{C2} & = & \hat{D}(\sqrt{1-{R}_{4}}\sqrt{f}\alpha \sqrt{{\eta }_{AB}{\eta }_{BC}}).\end{array}$$

In the case of an incomplete quantum channel among Alice, Bob, and Charlie, the success probability is displayed in Fig. 7(c). (The detailed analysis can be found in the Method section). Here, we assume ${q}_{+}=0.65,{q}_{-}=0.35$, and ${\eta }_{AB}={\eta }_{BC}=0.8$. In Fig. 7(c), the black solid line shows the optimal success probability of Bob and Charlie when an ideal situation is presented. In Fig. 7(c), the black dotted line (red dotted line) shows the success probability (optimal success probability) of the uncorrected (corrected) displacement operator in the Banaszek model. The black dotted line exhibits a discontinuity near ${\alpha }^{2}=1.3$, because the condition of the beam splitter changes rapidly. In the Banaszek model, the success probability decreases when the optical system is corrected.

**Figure 8:**
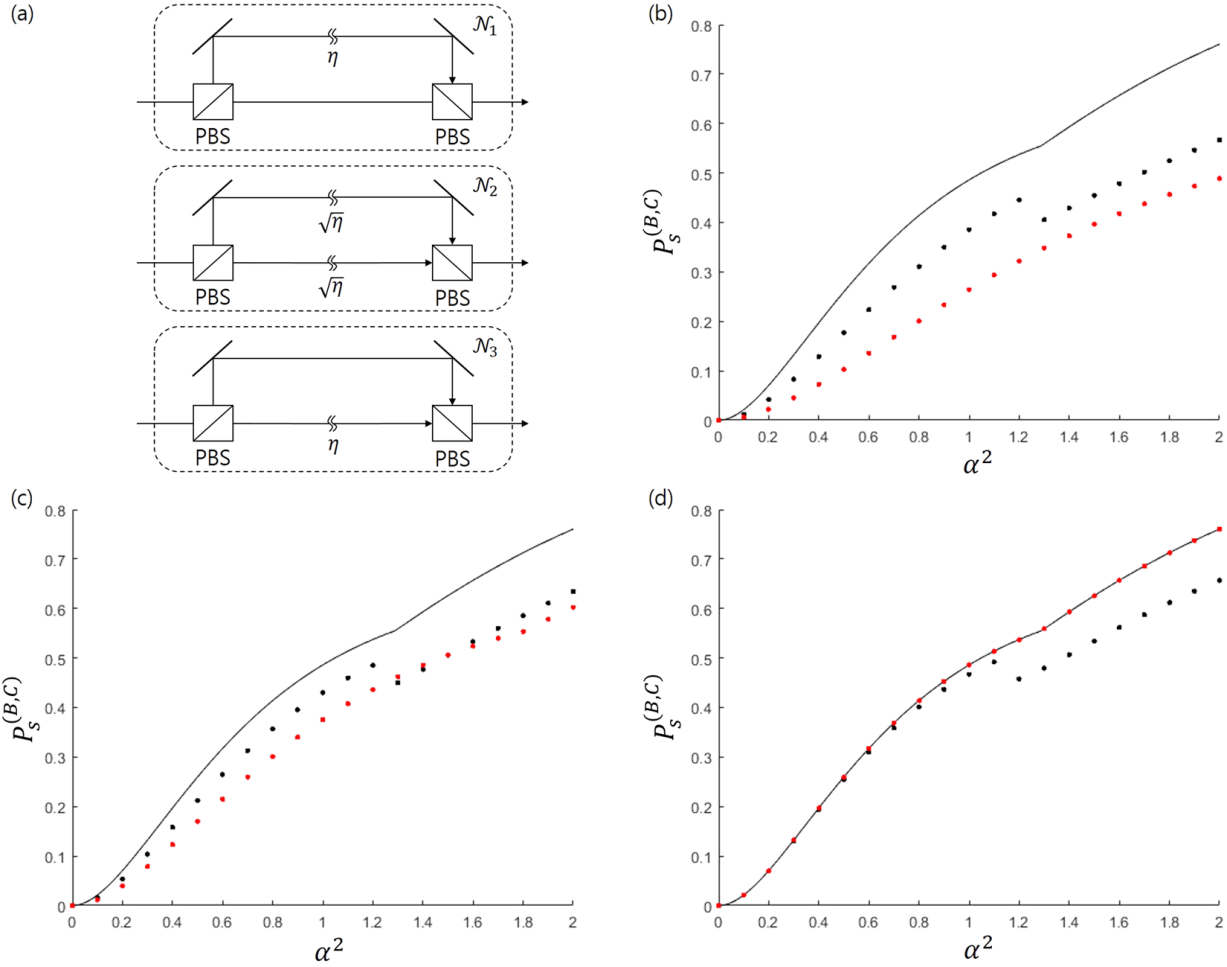
(**a**) The equivalent model of an incomplete quantum channel, which appears in the Huttner-like model. When the model exists between Alice and Bob (Bob and Charlie), we have $\eta ={\eta }_{AB}$ ($\eta ={\eta }_{BC}$). (**b**–**d**) Denote the success probability when the model ${{\mathscr{N}}}_{1}$, ${{\mathscr{N}}}_{2}$, and ${{\mathscr{N}}}_{3}$ are used. The solid line is the optimal success probability of the ideal case. The black(red) dotted line denotes the success probability when the uncorrected (corrected) displacement is considered. In (**b**–**d**), the BPSK signal is considered, with ${q}_{+}=0.65$.

*Imperfect Huttner*-*like model*. Since unlike the Banaszek model, the Huttner-like model combines a coherent signal and an auxiliary coherent state whose polarizations are othogonal, modeling an imcomplete quantum channel in the Huttner-like model is more difficult than in the Banaszek model. Figure 8(a) shows three quantum channels where photon loss is present. In the first model (${{\mathscr{N}}}_{1}$), only the coherent signal experiences photon loss. In the second model (${{\mathscr{N}}}_{2}$), an equal portion of photon loss exists in both the coherent signal and auxiliary coherent state. Meanwhile, In the third model (${{\mathscr{N}}}_{3}$), only an auxiliary coherent state experiences photon loss. The transmission rate of the beam splitter in ${{\mathscr{N}}}_{1}$ and ${{\mathscr{N}}}_{3}$ is ${\eta }_{AB}$. However, the transmission rate of the beam splitter in ${{\mathscr{N}}}_{2}$ is $\sqrt{{\eta }_{AB}}$. The incomplete quantum channel between Bob and Charlie has the same structure as the complete quantum channel between Alice and Bob, except for the transmission rate of the beam splitter.

Let us consider the BPSK signal $\{|\,+\,\alpha \rangle ,|\,-\,\alpha \rangle \}$ and the auxiliary coherent light $|\,-\,\alpha \rangle$. When the incomplete quantum channel is ${{\mathscr{N}}}_{1}$, Bob sends the post-measurement state $|\pm \sqrt{{R}_{1}}\alpha {\eta }_{AB}\rangle$ and the auxiliary coherent state $|-\,\sqrt{{R}_{2}}\alpha \rangle$ to Charlie. Then, Charlie obtains the coherent signal $|\pm \sqrt{{R}_{1}}\alpha {\eta }_{AB}{\eta }_{BC}\rangle$ and auxiliary coherent light $|-\sqrt{{R}_{2}}\alpha \rangle$. Bob and Charlie can discriminate Alice’s quantum state without error if their displacement operators are corrected in the following way:

26 $$\begin{array}{rcl}{\hat{D}}_{B1}\to {\hat{D}^{\prime} }_{B1} & = & \hat{D}(\sqrt{(1-{R}_{3})(1-{R}_{2})}\alpha -\sqrt{{R}_{3}(1-{R}_{1})}\alpha {\eta }_{AB}),\\ {\hat{D}}_{B2}\to {\hat{D}^{\prime} }_{B2} & = & \hat{D}(\sqrt{(1-{R}_{3})(1-{R}_{1})}\alpha {\eta }_{AB}-\sqrt{{R}_{3}(1-{R}_{2})}\alpha ),\\ {\hat{D}}_{C1}\to {\hat{D}^{\prime} }_{C1} & = & \hat{D}(\sqrt{(1-{R}_{4}){R}_{2}}\alpha -\sqrt{{R}_{4}{R}_{1}}\alpha {\eta }_{AB}{\eta }_{BC}),\\ {\hat{D}}_{C2}\to {\hat{D}^{\prime} }_{C2} & = & \hat{D}(\sqrt{(1-{R}_{4}){R}_{1}}\alpha {\eta }_{AB}{\eta }_{BC}-\sqrt{{R}_{4}{R}_{2}}\alpha ).\end{array}$$

when the incomplete quantum channel is ${{\mathscr{N}}}_{2}$, Bob combines the post-measurement state $|\pm \sqrt{{R}_{1}}\alpha \sqrt{{\eta }_{AB}}\rangle$ and the auxiliary coherent light $|-\sqrt{{R}_{2}}\alpha \sqrt{{\eta }_{AB}}\rangle$ and sends the signal to Charlie. Charlie receives the coherent signal $|\pm \sqrt{{R}_{1}}\alpha \sqrt{{\eta }_{AB}{\eta }_{BC}}\rangle$ and the auxiliary coherent light $|-\sqrt{{R}_{2}}\alpha \sqrt{{\eta }_{AB}{\eta }_{BC}}\rangle$ from Bob. If the displacement operators of Bob and Charlie are corrected in the following manner, it is possible to discriminate Alice’s quantum state without error:

27 $$\begin{array}{rcl}{\hat{D}}_{B1}\to {\hat{D}^{\prime} }_{B1} & = & \hat{D}(\sqrt{(1-{R}_{3})(1-{R}_{2})}\alpha \sqrt{{\eta }_{AB}}-\sqrt{{R}_{3}(1-{R}_{1})}\alpha \sqrt{{\eta }_{AB}}),\\ {\hat{D}}_{B2}\to {\hat{D}^{\prime} }_{B2} & = & \hat{D}(\sqrt{(1-{R}_{3})(1-{R}_{1})}\alpha \sqrt{{\eta }_{AB}}-\sqrt{{R}_{3}(1-{R}_{2})}\alpha \sqrt{{\eta }_{AB}}),\\ {\hat{D}}_{C1}\to {\hat{D}^{\prime} }_{C1} & = & \hat{D}(\sqrt{(1-{R}_{4}){R}_{2}}\alpha \sqrt{{\eta }_{AB}{\eta }_{BC}}-\sqrt{{R}_{4}{R}_{1}}\alpha \sqrt{{\eta }_{AB}{\eta }_{BC}}),\\ {\hat{D}}_{C2}\to {\hat{D}^{\prime} }_{C2} & = & \hat{D}(\sqrt{(1-{R}_{4}){R}_{1}}\alpha \sqrt{{\eta }_{AB}{\eta }_{BC}}-\sqrt{{R}_{4}{R}_{2}}\alpha \sqrt{{\eta }_{AB}{\eta }_{BC}}).\end{array}$$

when the incomplete quantum channel is ${{\mathscr{N}}}_{3}$, Bob combines the post-measurement state $|\pm \sqrt{{R}_{1}}\alpha \rangle$ and auxiliary coherent light $|-\sqrt{{R}_{2}}\alpha {\eta }_{AB}\rangle$. And Bob sends it to Charlie. Then, from Bob, Charlie receives coherent signal and auxiliary coherent light $|-\sqrt{{R}_{2}}\alpha {\eta }_{AB}{\eta }_{BC}\rangle$. If displacement operators of Bob and Charlie are corrected in the following manner, they can discriminate Alice’s quantum state without error:

28 $$\begin{array}{rcl}{\hat{D}}_{B1}\to {\hat{D}^{\prime} }_{B1} & = & \hat{D}(\sqrt{(1-{R}_{3})(1-{R}_{2})}\alpha {\eta }_{AB}-\sqrt{{R}_{3}(1-{R}_{1})}\alpha ),\\ {\hat{D}}_{B2}\to {\hat{D}^{\prime} }_{B2} & = & \hat{D}(\sqrt{(1-{R}_{3})(1-{R}_{1})}\alpha -\sqrt{{R}_{3}(1-{R}_{2})}\alpha {\eta }_{AB}),\\ {\hat{D}}_{C1}\to {\hat{D}^{\prime} }_{C1} & = & \hat{D}(\sqrt{(1-{R}_{4}){R}_{2}}\alpha {\eta }_{AB}{\eta }_{BC}-\sqrt{{R}_{4}{R}_{1}}\alpha ),\\ {\hat{D}}_{C2}\to {\hat{D}^{\prime} }_{C2} & = & \hat{D}(\sqrt{(1-{R}_{4}){R}_{1}}\alpha -\sqrt{{R}_{4}{R}_{2}}\alpha {\eta }_{AB}{\eta }_{BC}).\end{array}$$

Figure 8 shows the success probability of the quantum channels ${{\mathscr{N}}}_{i}(i\in \{1,2,3\})$. (A detailed analysis can be found in the Method section). The solid line represents the success probability of ideal quantum channels. The black dotted line (red dotted line) is the success probability of the corrected (uncorrected) case in the Huttner-like model. In ${{\mathscr{N}}}_{2}$ and ${{\mathscr{N}}}_{3}$, unlike the ideal case, the the success probability of the uncorrected case depends on *R*_2_. In Fig. 8(c,d), the black dotted line shows the largest case for ${R}_{2}\in [0,1]$. Similar to the Banaszek model, Fig. 8(b) shows that the success probability of the corrected case is smaller than that of the uncorrected case. Figure 8(b,c) show that the difference between the corrected and uncorrected cases diminishes. However, Fig. 8(d) shows that the success probability of the corrected case is larger than that of the uncorrected case. In addition, the success probability of the corrected case is identical to that of the ideal case.

In fact, the case where photon loss occurs can be understood as the case of existence of eavesdroppers. Let us suppose that there are eavesdroppers David and Eve. In that case, David tries to eavesdrop between Alice and Bob, but Eve tries to eavesdrop between Bob and Charlie. When David and Eve extract a maximum information from the coherent signal, it can be understood as ${{\mathscr{N}}}_{1}$ channel and the optimal success probability for the no-correction model diminishes rapidly. Therefore, Alice, Bob, and Charlie can detect the existence of the eavesdroppers David and Eve. Meanwhile, if David and Eve attempt to extract information from the auxiliary light, which can be understood as the case of ${{\mathscr{N}}}_{3}$ channel, the optimal success probability of the no-correction model diminishes slightly. In that case, Alice, Bob, and Charlie hardly notice the existence of eavesdroppers David and Eve. However, David and Eve cannot obtain any information from the coherent signal.

### Comparison with quantum probabilistic cloning scheme

In this section, we compare the sequential state discrimination strategy with the strategy of quantum probabilistic cloning. When elements of a set of pure states are not orthogonal to each other, there is no unitary operation to copy a pure state of the set^51^. However, there is a quantum operation to make a probabilistic copy of a pure state out of non-orthogonal pure states, which was first suggested by Duan and Guo^52^. Based on this idea, a method exists to copy an unknown coherent state with a certain probability. Quantum probabilistic cloning strategy of coherent states needs processes such as $|\alpha \rangle \otimes |0\rangle \to |\sqrt{2}\alpha \rangle \otimes |0\rangle \to |\alpha \rangle \otimes |\alpha \rangle$. Since the process of $|\sqrt{2}\alpha \rangle \otimes |0\rangle \to |\alpha \rangle \otimes |\alpha \rangle$ can be easily obtained using 50/50 beam splitter, one should find a method to increase the amplitude such as $|\alpha \rangle \otimes |0\rangle \to |\sqrt{2}\alpha \rangle \otimes |0\rangle$. Ho *et al*.^42^ described a method of probabilistic amplification of the coherent state using a controlled-Z gate^42^. According to J. Ho *et al*.^42^, the success probability of the process such that $|\alpha \rangle \to |g\alpha \rangle \simeq N^{\prime} (|0\rangle +g\alpha |1\rangle )$ is given by

29 $${P}_{clone}\simeq \frac{{N}^{^{\prime} 2}}{3}\frac{1}{1+3{g}^{2}}(1+{g}^{2}|\alpha {|}^{2}).$$

**Figure 9:**
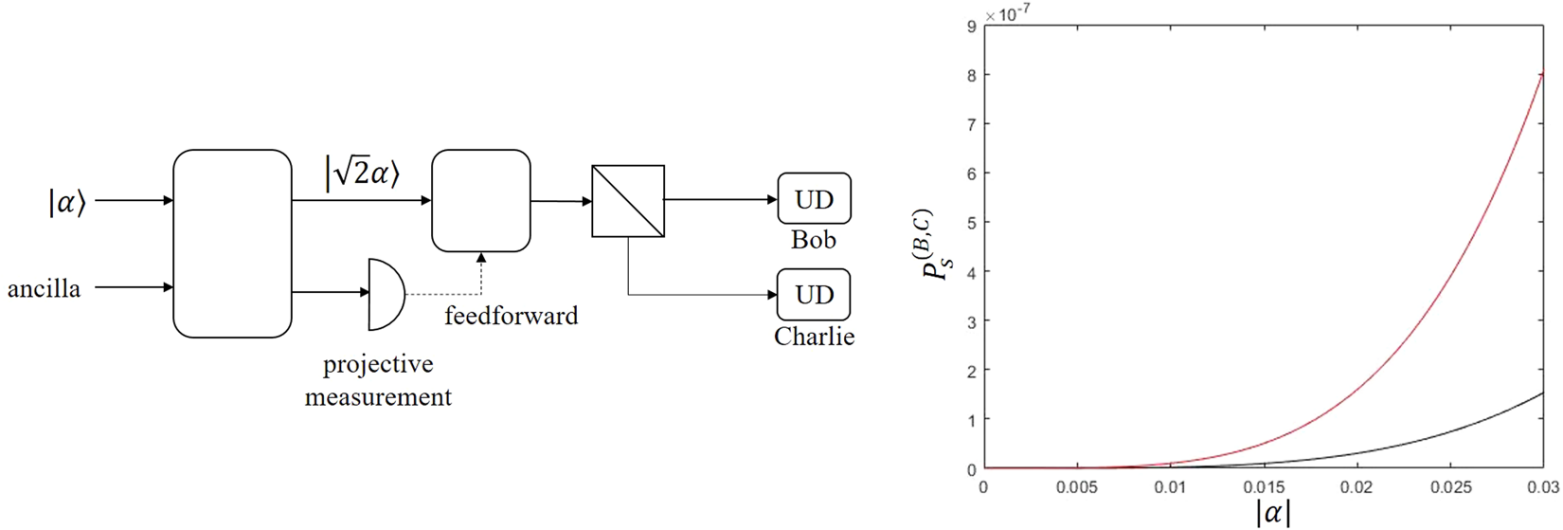
(**a**) The strategy using quantum probabilistic cloning^42^. Here, Bob and Charlie locally use optimal unambiguous discrimination. (**b**) The success probability of sequential state discrimination and quantum probabilistic cloning. The red solid line (black solid line) denotes the success probability of ideal sequential state discrimination (ideal quantum probabilistic cloning).

Figure 9(a) describes the state discrimination strategy of multi-parties using a quantum probabilistic cloning strategy. Alice prepares a coherent state $\alpha \in \{|+|\alpha |\rangle ,|-|\alpha |\rangle \}$ out of two coherent states $\{|+|\alpha |\rangle ,|-|\alpha |\rangle \}$, with equal prior probability. The coherent state $|\alpha \rangle$ goes through an optical controlled-Z gate, with the pure state (ancilla) in a logical basis. Ancilla is measured using the projective measurement. When quantum probabilistic cloning succeeds, one can obtain $|\sqrt{\alpha }\rangle (g=\sqrt{2})$ using the optical controlled-Z gate. Then, we can have $|\alpha \rangle \otimes |\alpha \rangle$ when $|\sqrt{2}\alpha \rangle$ is transmitted through the 50/50 BS. The state $|\alpha \rangle \otimes |\alpha \rangle$ is sent to Bob and Charlie. They should then perform an optimal discrimination on the phase of $|\alpha \rangle$, without an error. The probability, that Bob and Charlie optimally discriminate the quantum state, is obtained as:

30 $${P}_{clone}^{(B,C)}\simeq \frac{{e}^{-2|\alpha {|}^{2}}}{21}(1+2|\alpha {|}^{2}){(1-{e}^{-2|\alpha {|}^{2}})}^{2}.$$

The result of J. Ho *et al*.^42^ indicates that as |*α*| increases, *g* becomes smaller than the theoretical value. Therefore, if |*α*| is not very small, the success probability of quantum probabilistic cloning strategy is less than Eq. (14). Figure 9(b) shows the success probabilities of sequential state discrimination and quantum probabilistic cloning strategies when the quantum channel and detector are perfect. In this figure, sequential state discrimination strategy produces better success probability than the quantum probabilistic cloning strategy. In a realistic situation, the success probability of the sequential state discrimination strategy can be much larger than that of the quantum probabilistic cloning strategy.

## Discussion

In this report, we proposed optical designs for sequential state discrimination of two coherent states. We showed that the proposed Banaszek model and Huttner-like model can optimally perform sequential state discrimination. It should be noted that the two models use neither electric feedback nor complicated interactions, and are easier to implement than receivers for minimum error discrimination^35–41,44^. Moreover, since the Huttner-like model combines Alice’s coherent state with auxiliary light, it can provide a secure QKD to Alice, Bob, and Charlie.

Furthermore, we compared our strategy of sequential state discrimination to that of probabilistic quantum cloning. The probabilistic quantum cloning of coherent states includes a process that conditionally amplifies the amplitude of unknown coherent states. In addition, the amplification process can be applied only to coherent states with a weak amplitude. However, sequential state discrimination can be used in coherent states with any amplitude. In addition, the success probability of sequential state discrimination is larger than that of probabilistic quantum cloning.

Here, we considered sequential state discrimination of two coherent states. It is natural to extend our result to the case of *N* coherent states. Unfortunately, up to now, optimal unambiguous discrimination for *N* coherent states has not been shown. Even though F. E. Becerra *et al*.^47^ proposed unambiguous discrimination of four symmetric coherent states, the design does not provide optimal success probabilty^53^. Therefore, a deeper understanding of optimal unambiguous discrimination for *N* coherent states is needed.

## Methods

### Mathematical derivation of optimal success probability

In this section, we derive the optimal success probability. The derivation can be found in the result of M. Namkung *et al*.^28,30^. Since $({\alpha ^{\prime} }_{1},{\alpha ^{\prime} }_{2})$ exists on the tangential point between a plane ${P}_{s}=({q}_{1}{\alpha }_{1}){\alpha ^{\prime} }_{1}+({q}_{2}{\alpha }_{2}){\alpha ^{\prime} }_{2}$ and a surface $g({\alpha ^{\prime} }_{1},{\alpha ^{\prime} }_{2})=(1-{\alpha ^{\prime} }_{1})(1-{\alpha ^{\prime} }_{2})-s{^{\prime} }^{2}$, $({\alpha ^{\prime} }_{1},{\alpha ^{\prime} }_{2})$ can be obtained from the following condition as:

31 $$(\tfrac{\partial {P}_{s}^{(B,C)}}{\partial {\alpha ^{\prime} }_{1}},\tfrac{\partial {P}_{s}^{(B,C)}}{\partial {\alpha ^{\prime} }_{2}})=\lambda (\tfrac{\partial g}{\partial {\alpha ^{\prime} }_{1}},\tfrac{\partial g}{\partial {\alpha ^{\prime} }_{2}}),\,\lambda \in {\mathbb{R}}\backslash \{0\}\leftrightarrow \tfrac{\partial {P}_{s}^{(B,C)}/\partial {\alpha ^{\prime} }_{1}}{\partial {P}_{s}^{(B,C)}/\partial {\alpha ^{\prime} }_{2}}=\tfrac{\partial g/\partial {\alpha ^{\prime} }_{1}}{\partial g/\partial {\alpha ^{\prime} }_{2}}$$

Eq. (31) yields the following relation:

32 $$\begin{array}{rcl}(1-{\alpha ^{\prime} }_{2}) & = & \frac{{q}_{1}{\alpha }_{1}}{{q}_{2}{\alpha }_{2}}(1-{\alpha ^{\prime} }_{1})\\ (1-{\alpha ^{\prime} }_{1}) & = & \frac{{q}_{2}{\alpha }_{2}}{{q}_{1}{\alpha }_{1}}(1-{\alpha ^{\prime} }_{2})\end{array}$$

By Eq. (32) and $g({\alpha ^{\prime} }_{1},{\alpha ^{\prime} }_{2})=0$, we have following equations:

33 $$\begin{array}{rcl}{\alpha ^{\prime} }_{1} & = & 1-s^{\prime} \sqrt{\frac{{q}_{2}{\alpha }_{2}}{{q}_{1}{\alpha }_{1}}}=1-\frac{s}{\sqrt{(1-{\alpha }_{1})(1-{\alpha }_{2})}}\sqrt{\frac{{q}_{2}{\alpha }_{2}}{{q}_{1}{\alpha }_{1}}}\\ {\alpha ^{\prime} }_{2} & = & 1-s^{\prime} \sqrt{\frac{{q}_{1}{\alpha }_{1}}{{q}_{2}{\alpha }_{2}}}=1-\frac{s}{\sqrt{(1-{\alpha }_{1})(1-{\alpha }_{2})}}\sqrt{\frac{{q}_{1}{\alpha }_{1}}{{q}_{2}{\alpha }_{2}}}\end{array}$$

when $0 < {\alpha ^{\prime} }_{i} < 1-s{^{\prime} }^{2}(\forall i\in \{1,2\})$ is fulfilled, the object function ${P}_{s}^{(B,C)}$ is given by

34 $$\begin{array}{rcl}{P}_{s}^{(B,C)} & = & {q}_{1}{\alpha }_{1}\{1-\tfrac{s}{\sqrt{(1-{\alpha }_{1})(1-{\alpha }_{2})}}\sqrt{\tfrac{{q}_{2}{\alpha }_{2}}{{q}_{1}{\alpha }_{1}}}\}+{q}_{2}{\alpha }_{2}\{1-\tfrac{s}{\sqrt{(1-{\alpha }_{1})(1-{\alpha }_{2})}}\sqrt{\tfrac{{q}_{1}{\alpha }_{1}}{{q}_{2}{\alpha }_{2}}}\}\\  & = & {q}_{1}{\alpha }_{1}+{q}_{2}{\alpha }_{2}-2s\sqrt{\tfrac{{q}_{1}{q}_{2}{\alpha }_{1}{\alpha }_{2}}{(1-{\alpha }_{1})(1-{\alpha }_{2})}}\end{array}$$

The inequality, which is equivalent to $0 < {\alpha ^{\prime} }_{i} < 1-s{^{\prime} }^{2}$, can be given as follows:

35 $$\begin{array}{ll}{\alpha }_{2} < \frac{{\alpha }_{1}(1-{\alpha }_{1})}{x+{\alpha }_{1}(1-{\alpha }_{1})}, & x={s}^{2}\frac{{q}_{2}}{{q}_{1}}\\ {\alpha }_{1} < \frac{{\alpha }_{2}(1-{\alpha }_{2})}{y+{\alpha }_{2}(1-{\alpha }_{2})}, & y={s}^{2}\frac{{q}_{1}}{{q}_{2}}\end{array}$$

If ${\alpha ^{\prime} }_{1}=1-s{^{\prime} }^{2},{\alpha ^{\prime} }_{2}=0$, the success probability can be analytically obtained:

36 $${P}_{s}^{(B,C)}={q}_{1}{\alpha }_{1}\{1-\frac{{s}^{2}}{(1-{\alpha }_{1})(1-{\alpha }_{2})}\}\le {q}_{1}{\alpha }_{1}\{1-\frac{{s}^{2}}{1-{\alpha }_{1}}\}\le {q}_{1}{(1-s)}^{2}$$

In the first inequality, the equality condition is ${\alpha }_{1}=0$, which implies that Bob discriminates only $|{\psi }_{1}\rangle$. The second inequality can be analytically obtained from the condition $\partial {P}_{s}^{(B,C)}/\partial {\alpha }_{1}=0$.

When ${\alpha ^{\prime} }_{2}=1-s{^{\prime} }^{2},{\alpha ^{\prime} }_{1}=0$, the success probability can be found as

37 $${P}_{s}^{(B,C)}\le {q}_{2}{(1-s)}^{2}$$

By combining Eq. (36) with Eq. (37), we find ${P}_{s}^{(B,C)\ast }=\,{\rm{\max }}\,\{{q}_{1},{q}_{2}\}{(1-s)}^{2}$.

### Analytic optimal success probability and optimal designs in the case of equal prior probabilities

In the main text, we saw that the Banaszek model or Huttner-like model can faciliate an optimal success probability. Even though the success probability of two pure states with arbitrary prior probability cannot be analytically found, the success probability of two pure states with identical prior probability can be analytically obtained^26^. In this section, we show that when the prior probabilities are identical, the optimal success probability of the Banaszek model and Huttner-like model is equal to the result of J. A. Bergou *et al*.^26^.

**Figure 10:**
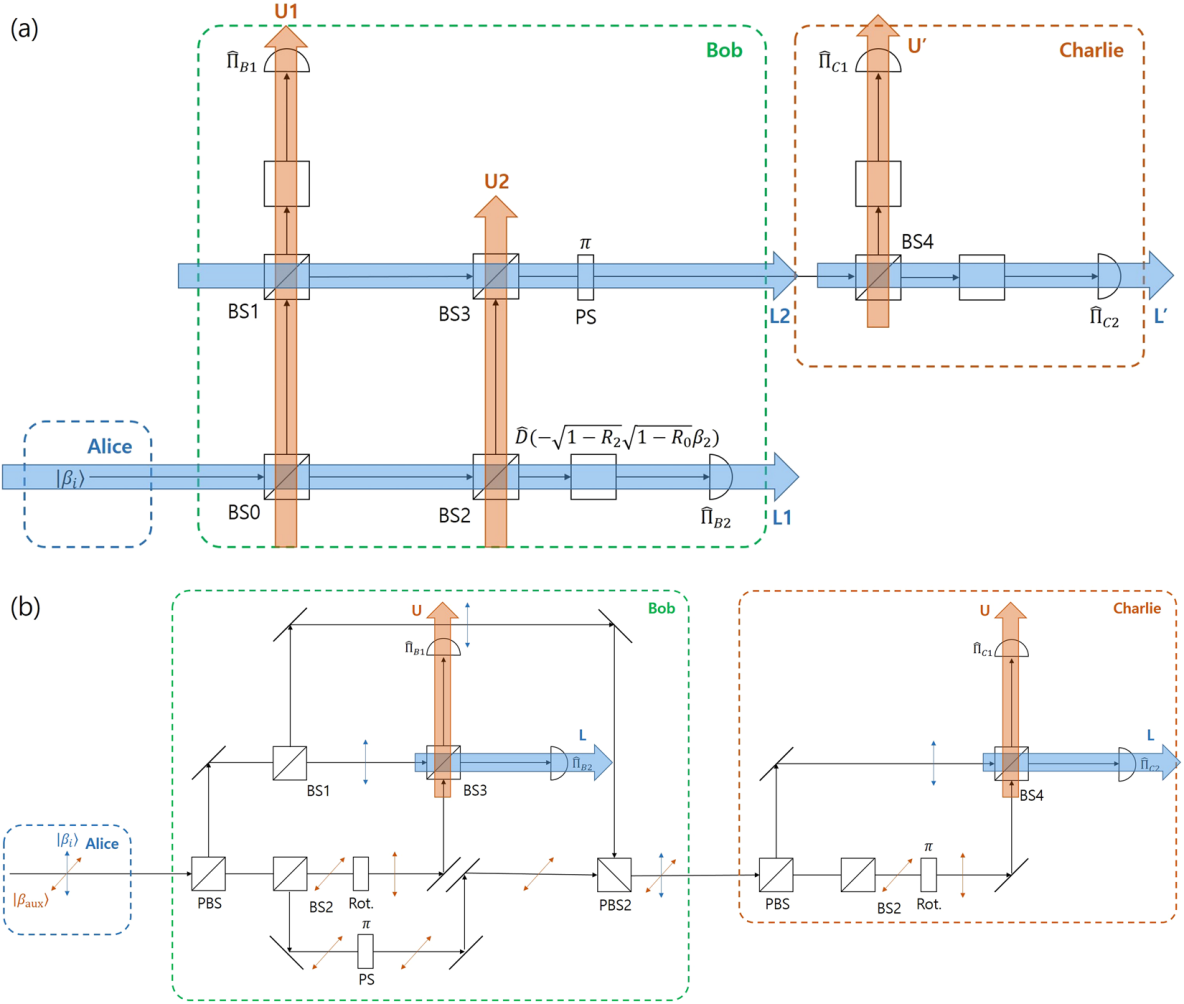
The path in Banaszek model and Huttner-like model. In these examples, we use 50/50 beam splitters.

Figure 10(a) shows the Banaszek model which performs sequential state discrimination with an optimal success probability in the result of J. A. Bergou *et al*.^26^. The model consists of only a 50/50 beam splitters (BS). In the Banaszek model, let us denote the paths to be L1(Left1), U1(Up1), L2(Left2), and U2(Up2), respectively. Each beam splitter performs the following transformation ${|\beta \rangle }_{{\rm{L}}}\otimes {|0\rangle }_{{\rm{U}}}\mathop{\longrightarrow }\limits^{{\rm{BS}}}{|\beta /\sqrt{2}\rangle }_{{\rm{L}}}\otimes {|\beta /\sqrt{2}\rangle }_{{\rm{U}}}$, ${|0\rangle }_{{\rm{L}}}\otimes {|\beta \rangle }_{{\rm{U}}}\mathop{\longrightarrow }\limits^{{\rm{BS}}}{|-\beta /\sqrt{2}\rangle }_{{\rm{L}}}\otimes {|\beta /\sqrt{2}\rangle }_{{\rm{U}}}$. For the beam splitter used in the Banaszek model, the input state transforms in the following way:

38 $$\begin{array}{l}{|{\beta }_{i}\rangle }_{{\rm{L}}1}\otimes {|0\rangle }_{{\rm{U}}1}\otimes {|0\rangle }_{{\rm{L}}2}\otimes {|0\rangle }_{{\rm{U}}2}\\ \,\mathop{\longrightarrow }\limits^{{\rm{BS0}}}\,{|\frac{{\beta }_{i}}{\sqrt{2}}\rangle }_{{\rm{L}}1}\otimes {|\frac{{\beta }_{i}}{\sqrt{2}}\rangle }_{{\rm{U}}1}\otimes {|0\rangle }_{{\rm{L}}2}\otimes {|0\rangle }_{{\rm{U}}2}\\ \,\mathop{\longrightarrow }\limits^{{\rm{BS1}}}\,{|\frac{{\beta }_{i}}{\sqrt{2}}\rangle }_{{\rm{L}}1}\otimes {|\frac{{\beta }_{i}}{2}\rangle }_{{\rm{U}}1}\otimes {|-\frac{{\beta }_{i}}{2}\rangle }_{{\rm{L}}2}\otimes {|0\rangle }_{{\rm{U}}2}\\ \,\mathop{\longrightarrow }\limits^{{\rm{BS2}}}\,{|\frac{{\beta }_{i}}{2}\rangle }_{{\rm{L}}1}\otimes {|\frac{{\beta }_{i}}{2}\rangle }_{{\rm{U}}1}\otimes {|-\frac{{\beta }_{i}}{2}\rangle }_{{\rm{L}}2}\otimes {|\frac{{\beta }_{i}}{2}\rangle }_{{\rm{U}}2}\end{array}$$

when the displacement operator $\hat{D}{(-{\beta }_{2}/\mathrm{2)}}_{{\rm{L}}1}\otimes \hat{D}{(-{\beta }_{1}/\mathrm{2)}}_{{\rm{U}}1}$ is applied, the following state is obtained:

39 $$\begin{array}{c}{|\frac{{\beta }_{i}}{2}\rangle }_{{\rm{L}}1}\otimes {|\frac{{\beta }_{i}}{2}\rangle }_{{\rm{U}}1}\otimes {|-\frac{{\beta }_{i}}{2}\rangle }_{{\rm{L}}2}\otimes {|\frac{{\beta }_{i}}{2}\rangle }_{{\rm{U}}2}\\ \,\mathop{\longrightarrow }\limits^{\hat{D}{(-\frac{{\beta }_{2}}{2})}_{{\rm{L}}1}\otimes \hat{D}{(-\frac{{\beta }_{1}}{2})}_{{\rm{U}}1}}\,{|\frac{1}{2}({\beta }_{i}-{\beta }_{2})\rangle }_{{\rm{L}}1}\otimes {|\frac{1}{2}({\beta }_{i}-{\beta }_{1})\rangle }_{{\rm{U}}1}\otimes {|-\frac{{\beta }_{i}}{2}\rangle }_{{\rm{L}}2}\otimes {|\frac{{\beta }_{i}}{2}\rangle }_{{\rm{U}}2}\end{array}$$

Finally, passing through BS3 and PS (phase shifter), the state becomes the final state:

40 $$\begin{array}{l}{|\frac{1}{2}({\beta }_{i}-{\beta }_{2})\rangle }_{{\rm{L}}1}\otimes {|\frac{1}{2}({\beta }_{i}-{\beta }_{1})\rangle }_{{\rm{U}}1}\otimes {|-\frac{{\beta }_{i}}{2}\rangle }_{{\rm{L}}2}\otimes {|\frac{{\beta }_{i}}{2}\rangle }_{{\rm{U}}2}\\ \,\mathop{\longrightarrow }\limits^{{\rm{BS3}}}\,{|\frac{1}{2}({\beta }_{i}-{\beta }_{2})\rangle }_{{\rm{L}}1}\otimes {|\frac{1}{2}({\beta }_{i}-{\beta }_{1})\rangle }_{{\rm{U}}1}\otimes {|-\frac{{\beta }_{i}}{\sqrt{2}}\rangle }_{{\rm{L}}2}\otimes {|0\rangle }_{{\rm{U}}2}\\ \,\mathop{\longrightarrow }\limits^{{\rm{PS}}}\,{|\frac{1}{2}({\beta }_{i}-{\beta }_{2})\rangle }_{{\rm{L}}1}\otimes {|\frac{1}{2}({\beta }_{i}-{\beta }_{1})\rangle }_{{\rm{U}}1}\otimes {|\frac{{\beta }_{i}}{\sqrt{2}}\rangle }_{{\rm{L}}2}\otimes {|0\rangle }_{{\rm{U}}2}\end{array}$$

The probability of a conclusive result in Bob’s photon detectors is given by

41 $$\begin{array}{l}{{\rm{\Pr }}}_{{\rm{B}}}[(\mathrm{off},\mathrm{on})|{\beta }_{1}]\\ \begin{array}{rcl} & = & {\rm{Tr}}\{{|\frac{1}{2}({\beta }_{1}-{\beta }_{2})\rangle }_{{\rm{L}}1}{\langle \frac{1}{2}({\beta }_{1}-{\beta }_{2})|\otimes |0\rangle }_{{\rm{U}}1}{\langle 0|\otimes |-\frac{{\beta }_{1}}{2}\rangle }_{{\rm{L}}2}\\  &  & \times \,{\langle -\frac{{\beta }_{1}}{2}|\otimes |0\rangle }_{{\rm{U}}2}\langle 0|\,{\hat{{\rm{\Pi }}}}_{{\rm{B}}1}^{(\mathrm{on})}\otimes {\hat{{\rm{\Pi }}}}_{{\rm{B}}2}^{(\mathrm{off})}\otimes {{\mathbb{I}}}_{{\rm{L}}2}\otimes {{\mathbb{I}}}_{{\rm{U}}2}\}\\  & = & 1-\exp (-\frac{1}{4}|{\beta }_{1}-{\beta }_{2}{|}^{2})\end{array}\\ {{\rm{\Pr }}}_{{\rm{B}}}[(\mathrm{on},\mathrm{off})|{\beta }_{2}]\\ \begin{array}{rcl} & = & {\rm{Tr}}\{{|0\rangle }_{{\rm{L}}1}{\langle 0|\otimes |\frac{1}{2}({\beta }_{2}-{\beta }_{1})\rangle }_{{\rm{U}}1}{\langle \frac{1}{2}({\beta }_{2}-{\beta }_{1})|\otimes |-\frac{{\beta }_{2}}{2}\rangle }_{{\rm{L}}2}\\  &  & \times \,{\langle -\frac{{\beta }_{2}}{2}|\otimes |0\rangle }_{{\rm{U}}2}\langle 0|\,{\hat{{\rm{\Pi }}}}_{{\rm{B}}1}^{(\mathrm{off})}\otimes {\hat{{\rm{\Pi }}}}_{{\rm{B}}2}^{(\mathrm{on})}\otimes {{\mathbb{I}}}_{{\rm{L}}2}\otimes {{\mathbb{I}}}_{{\rm{U}}2}\}\\  & = & 1-\exp (-\frac{1}{4}|{\beta }_{1}-{\beta }_{2}{|}^{2})\end{array}\end{array}$$

Meanwhile, the post-measurement state of Bob is obtained as:

42 $$\begin{array}{c}{{\rm{Tr}}}_{{\rm{L1}},{\rm{U1}},{\rm{U2}}}\{{|\frac{1}{2}({\beta }_{i}-{\beta }_{2})\rangle }_{{\rm{L1}}}{\langle \frac{1}{2}({\beta }_{i}-{\beta }_{2})|\otimes |\frac{1}{2}({\beta }_{i}-{\beta }_{1})\rangle }_{{\rm{U1}}}\\ \,\times \,{\langle \frac{1}{2}({\beta }_{i}-{\beta }_{1})|\otimes |\frac{{\beta }_{i}}{\sqrt{2}}\rangle }_{{\rm{L}}2}{\langle \frac{{\beta }_{i}}{\sqrt{2}}|\otimes |0\rangle }_{{\rm{U2}}}0\}=|\frac{{\beta }_{i}}{\sqrt{2}}\rangle {\langle \frac{{\beta }_{i}}{\sqrt{2}}|}_{{\rm{L}}2}\end{array}$$

when the post-measurement state of Bob is $|{\beta }_{i}/\sqrt{2}\rangle$, this state transforms into the following form (Here, ${\rm{L}}2={\rm{L}}^{\prime}$):

43 $$\begin{array}{c}{|\frac{{\beta }_{i}}{\sqrt{2}}\rangle }_{{\rm{L}}^{\prime} }\otimes {|0\rangle }_{{\rm{U}}^{\prime} }\\ \,\mathop{\longrightarrow }\limits^{{\rm{BS4}}}\,{|\frac{{\beta }_{i}}{2}\rangle }_{{\rm{L}}^{\prime} }\otimes {|\frac{{\beta }_{i}}{2}\rangle }_{{\rm{U}}^{\prime} }\\ \,\mathop{\longrightarrow }\limits^{\hat{D}{(-\frac{{\beta }_{2}}{2})}_{{\rm{L}}^{\prime} }\otimes \hat{D}{(-\frac{{\beta }_{1}}{2})}_{{\rm{U}}^{\prime} }}\,{|\frac{1}{2}({\beta }_{i}-{\beta }_{2})\rangle }_{{\rm{L}}^{\prime} }\otimes {|\frac{1}{2}({\beta }_{i}-{\beta }_{1})\rangle }_{{\rm{U}}^{\prime} }\end{array}$$

Therefore, the probability of a conclusive result in Charlie’s photon detectors is as follows:

44 $$\begin{array}{l}{{\rm{\Pr }}}_{{\rm{C}}}[({\rm{off}},{\rm{on}})|{\beta }_{1}]\\ \begin{array}{rcl} & = & {\rm{Tr}}\{{|\frac{1}{2}({\beta }_{1}-{\beta }_{2})\rangle }_{{\rm{L}}^{\prime} }{\langle \frac{1}{2}({\beta }_{1}-{\beta }_{2})|\otimes |0\rangle }_{{\rm{U}}^{\prime} }\langle 0|\,{\hat{{\rm{\Pi }}}}_{{\rm{B}}1}^{({\rm{off}})}\otimes {\hat{{\rm{\Pi }}}}_{{\rm{B}}2}^{({\rm{on}})}\}\\  & = & 1-\exp (-\frac{1}{4}|{\beta }_{1}-{\beta }_{2}{|}^{2})\end{array}\\ {{\rm{\Pr }}}_{{\rm{C}}}[({\rm{on}},{\rm{off}})|{\beta }_{2}]\\ \begin{array}{rcl} & = & {\rm{Tr}}\{{|0\rangle }_{{\rm{L}}^{\prime} }{\langle 0|\otimes |\frac{1}{2}({\beta }_{2}-{\beta }_{1})\rangle }_{{\rm{U}}^{\prime} }\langle \frac{1}{2}({\beta }_{2}-{\beta }_{1})|\,{\hat{{\rm{\Pi }}}}_{{\rm{B}}1}^{({\rm{on}})}\otimes {\hat{{\rm{\Pi }}}}_{{\rm{B}}2}^{({\rm{off}})}\}\\  & = & 1-\exp (-\frac{1}{4}|{\beta }_{1}-{\beta }_{2}{|}^{2})\end{array}\end{array}$$

Then, the success probability that Bob and Charlie can perform sequential state discrimination is given by

45 $$\begin{array}{rcl}{P}_{s}^{(B,C)} & = & \frac{1}{2}{{\rm{\Pr }}}_{{\rm{B}}}[({\rm{off}},{\rm{on}})|{\beta }_{1}]\,{{\rm{\Pr }}}_{{\rm{C}}}[({\rm{off}},{\rm{on}})|{\beta }_{1}]\\  &  & +\,\frac{1}{2}{{\rm{\Pr }}}_{{\rm{C}}}[({\rm{off}},{\rm{on}})|{\beta }_{1}]\,{{\rm{\Pr }}}_{{\rm{C}}}[({\rm{on}},{\rm{off}})|{\beta }_{2}]\\  & = & {\{1-\exp (-\frac{1}{4}|{\beta }_{1}-{\beta }_{2}{|}^{2})\}}^{2}\end{array}$$

The optimal success probability is identical to the result of Bergou *et al*.^26^.

Next, we show that the Huttner-like model can provide the optimal success probability in the result of J. A. Bergou *et al*.^26^. The Huttner-like model is shown in Fig. 10(b). Suppose that Alice combines coherent light $|\pm \alpha \rangle$ in the vertical direction with auxiliary coherent light $|-\alpha \rangle$ and sends it to Bob. Then, in Bob’s beam splitter BS3, we can see the following transformation:

46 $$\begin{array}{lll}{|+\frac{\alpha }{\sqrt{2}}\rangle }_{{\rm{L}}}\otimes {|-\frac{\alpha }{\sqrt{2}}\rangle }_{{\rm{U}}} & \mathop{\longrightarrow }\limits^{{\rm{BS3}}} & {|0\rangle }_{{\rm{L}}}\otimes {|+\alpha \rangle }_{{\rm{U}}}\\ {|-\frac{\alpha }{\sqrt{2}}\rangle }_{{\rm{L}}}\otimes {|-\frac{\alpha }{\sqrt{2}}\rangle }_{{\rm{U}}} & \mathop{\longrightarrow }\limits^{{\rm{BS3}}} & {|-\alpha \rangle }_{{\rm{L}}}\otimes {|0\rangle }_{{\rm{U}}}\end{array}$$

The probability of a conclusive result in Bob’s photon detectors is given by

47 $$\begin{array}{rcl}{{\rm{\Pr }}}_{{\rm{B}}}[({\rm{off}},{\rm{on}})|\,+\,\alpha ] & = & {\rm{Tr}}\{{|0\rangle }_{{\rm{L}}}\langle 0|\otimes {|+\alpha \rangle }_{{\rm{U}}}\langle +\alpha |\,{\hat{{\rm{\Pi }}}}_{{\rm{B}}1}^{({\rm{off}})}\otimes {\hat{{\rm{\Pi }}}}_{{\rm{B}}2}^{({\rm{on}})}\}\\  & = & 1-\exp (-|\alpha {|}^{2})\\ {{\rm{\Pr }}}_{{\rm{B}}}[({\rm{on}},{\rm{off}})|\,-\,\alpha ] & = & {\rm{Tr}}\{{|-\alpha \rangle }_{{\rm{L}}}\langle -\alpha |\otimes {|0\rangle }_{{\rm{U}}}\langle 0|\,{\hat{{\rm{\Pi }}}}_{{\rm{B}}1}^{({\rm{on}})}\otimes {\hat{{\rm{\Pi }}}}_{{\rm{B}}2}^{({\rm{off}})}\}\\  & = & 1-\exp (-|\alpha {|}^{2})\end{array}$$

In BS4 of Charlie’s optical system, a similar transformation to Eq. (47) occurs. Therefore, we have ${{\rm{\Pr }}}_{{\rm{B}}}[({\rm{off}},{\rm{on}})|\,+\,\alpha ]={{\rm{\Pr }}}_{{\rm{C}}}[({\rm{off}},{\rm{on}})|\,+\,\alpha ]$, ${{\rm{\Pr }}}_{{\rm{B}}}[({\rm{on}},{\rm{off}})|\,-\,\alpha ]={{\rm{\Pr }}}_{{\rm{C}}}[({\rm{on}},{\rm{off}})|\,-\,\alpha ]$. Then, the success probability that Bob and Charlie can perform sequential state discrimination is obtained as

48 $$\begin{array}{rcl}{P}_{s}^{(B,C)} & = & \frac{1}{2}{{\rm{\Pr }}}_{{\rm{B}}}[({\rm{off}},{\rm{on}})|\,+\,\alpha ]\,{{\rm{\Pr }}}_{{\rm{C}}}[({\rm{off}},{\rm{on}})|\,+\,\alpha ]\\  &  & +\,\frac{1}{2}{{\rm{\Pr }}}_{{\rm{B}}}[({\rm{on}},{\rm{off}})|\,-\,\alpha ]\,{{\rm{\Pr }}}_{{\rm{C}}}[({\rm{on}},{\rm{off}})|\,-\,\alpha ]\\  & = & {\{1-\exp (-|\alpha {|}^{2})\}}^{2}\end{array}$$

We can see that the optimal success probability is identical to the result of Bergou *et al*.^26^.

### Analytic success probabilities of imperfect scenario

In this section, we determine the success probability in the case of an optical design with no-correction. Firstly, the success probability of the Banaszek model without correction is given by

49 $$\begin{array}{rcl}{P}_{s}^{(B,C)} & = & {q}_{+}{e}^{-{(\sqrt{{\eta }_{AB}}-1)}^{2}(1-{R}_{1}){R}_{0}{\alpha }^{2}}\{1-{e}^{-{(\sqrt{{\eta }_{AB}}+1)}^{2}(1-{R}_{2})(1-{R}_{0}){\alpha }^{2}}\}\\  &  & \times \,{e}^{-{(\sqrt{{\eta }_{AB}{\eta }_{BC}}-1)}^{2}{R}_{4}f{\alpha }^{2}}\{1-{e}^{-{(\sqrt{{\eta }_{AB}{\eta }_{BC}}+1)}^{2}(1-{R}_{4})f{\alpha }^{2}}\}\\  &  & +\,{q}_{-}\{1-{e}^{-{(\sqrt{{\eta }_{AB}}+1)}^{2}(1-{R}_{1}){R}_{0}{\alpha }^{2}}\}{e}^{-{(\sqrt{{\eta }_{AB}}-1)}^{2}(1-{R}_{2})(1-{R}_{0}){\alpha }^{2}}\\  &  & \times \,\{1-{e}^{-{(\sqrt{{\eta }_{AB}{\eta }_{BC}}+1)}^{2}{R}_{4}f{\alpha }^{2}}\}{e}^{-{(\sqrt{{\eta }_{AB}{\eta }_{BC}}-1)}^{2}(1-{R}_{4})f{\alpha }^{2}}\end{array}$$

Secondly, the success probability of the Huttner-like model without correction is obatained as:

(channel model: $${{\mathscr{N}}}_{1}$$)

50 $$\begin{array}{rcl}{P}_{s}^{(B,C)} & = & {q}_{+}{e}^{-{\alpha }^{2}{({\eta }_{AB}-1)}^{2}{R}_{3}(1-{R}_{1})}\{1-{e}^{-{\alpha }^{2}{({\eta }_{AB}+1)}^{2}(1-{R}_{3})(1-{R}_{1})}\}\\  &  & \times \,{e}^{-{\alpha }^{2}{({\eta }_{AB}{\eta }_{BC}-1)}^{2}{R}_{4}{R}_{1}}\{1-{e}^{-{\alpha }^{2}{({\eta }_{AB}{\eta }_{BC}+1)}^{2}(1-{R}_{4}){R}_{1}}\}\\  &  & +\,{q}_{-}\{1-{e}^{-{\alpha }^{2}{({\eta }_{AB}+1)}^{2}{R}_{3}(1-{R}_{1})}\}{e}^{-{\alpha }^{2}{({\eta }_{AB}-1)}^{2}(1-{R}_{3})(1-{R}_{1})}\\  &  & \times \,\{1-{e}^{-{\alpha }^{2}{({\eta }_{AB}{\eta }_{BC}+1)}^{2}{R}_{4}{R}_{1}}\}{e}^{-{\alpha }^{2}{({\eta }_{AB}{\eta }_{BC}-1)}^{2}(1-{R}_{4}){R}_{1}}\end{array}$$

(channel model: $${{\mathscr{N}}}_{2}$$)

51 $$\begin{array}{rcl}{P}_{s}^{(B,C)} & = & {q}_{+}{e}^{-{\alpha }^{2}{(\sqrt{{\eta }_{AB}}-\mathrm{1)}}^{2}{(\sqrt{{R}_{3}}\sqrt{1-{R}_{1}}-\sqrt{1-{R}_{3}}\sqrt{1-{R}_{2}})}^{2}}\\  &  & \times \,\{1-{e}^{-{\alpha }^{2}{(\sqrt{\mathrm{(1}-{R}_{3})}\sqrt{1-{R}_{1}}(\sqrt{{\eta }_{AB}}+\mathrm{1)}+\sqrt{{R}_{3}}\sqrt{1-{R}_{2}}(\sqrt{{\eta }_{AB}}-\mathrm{1))}}^{2}}\}\\  &  & \times \,{e}^{-{\alpha }^{2}{(\sqrt{{\eta }_{AB}{\eta }_{BC}}-\mathrm{1)}}^{2}{(\sqrt{{R}_{4}}\sqrt{{R}_{1}}-\sqrt{1-{R}_{4}}\sqrt{{R}_{2}})}^{2}}\\  &  & \times \,\{1-{e}^{-{\alpha }^{2}{(\sqrt{1-{R}_{4}}\sqrt{{R}_{1}}(\sqrt{{\eta }_{AB}{\eta }_{BC}}+\mathrm{1)}+\sqrt{{R}_{4}}\sqrt{{R}_{2}}(\sqrt{{\eta }_{AB}{\eta }_{BC}}-\mathrm{1))}}^{2}}\}\\  &  & +\,{q}_{-}\{1-{e}^{-{\alpha }^{2}{(\sqrt{{R}_{3}}\sqrt{1-{R}_{1}}(\sqrt{{\eta }_{AB}}+\mathrm{1)}+\sqrt{1-{R}_{3}}\sqrt{1-{R}_{2}}(\sqrt{{\eta }_{AB}}-\mathrm{1))}}^{2}}\}\\  &  & \times \,{e}^{-{\alpha }^{2}{(\sqrt{{\eta }_{AB}}-\mathrm{1)}}^{2}{(\sqrt{{R}_{3}}\sqrt{1-{R}_{2}}-\sqrt{1-{R}_{3}}\sqrt{1-{R}_{1}})}^{2}}\\  &  & \times \,{e}^{-{\alpha }^{2}{(\sqrt{{\eta }_{AB}{\eta }_{BC}}-\mathrm{1)}}^{2}{(\sqrt{{R}_{4}}\sqrt{{R}_{2}}-\sqrt{1-{R}_{4}}\sqrt{{R}_{1}})}^{2}}\\  &  & \times \,\{1-{e}^{-{\alpha }^{2}{(\sqrt{{R}_{4}}\sqrt{{R}_{1}}(\sqrt{{\eta }_{AB}{\eta }_{BC}}+\mathrm{1)}+\sqrt{1-{R}_{4}}\sqrt{{R}_{2}}(\sqrt{{\eta }_{AB}{\eta }_{BC}}-\mathrm{1))}}^{2}}\}\end{array}$$

(channel model: $${{\mathscr{N}}}_{3}$$)

52 $$\begin{array}{rcl}{P}_{s}^{(B,C)} & = & {q}_{+}{e}^{-{\alpha }^{2}{(1-{\eta }_{AB})}^{2}(1-{R}_{3})(1-{R}_{2})}\{1-{e}^{-{\alpha }^{2}{(2\sqrt{1-{R}_{3}}\sqrt{1-{R}_{1}}+\sqrt{{R}_{3}}\sqrt{1-{R}_{2}}({\eta }_{AB}-1))}^{2}}\}\\  &  & \times \,{e}^{-{\alpha }^{2}{(1-{\eta }_{AB}{\eta }_{BC})}^{2}(1-{R}_{4}){R}_{2}}\{1-{e}^{-{\alpha }^{2}{(2\sqrt{1-{R}_{4}}\sqrt{{R}_{1}}+\sqrt{{R}_{4}}\sqrt{{R}_{2}}({\eta }_{AB}{\eta }_{BC}-1))}^{2}}\}\\  &  & +\,{q}_{-}\{1-{e}^{-{\alpha }^{2}{(2\sqrt{{R}_{3}}\sqrt{1-{R}_{1}}+\sqrt{1-{R}_{3}}\sqrt{1-{R}_{2}}({\eta }_{AB}-1))}^{2}}\}{e}^{-{\alpha }^{2}{R}_{3}(1-{R}_{2}){(1-{\eta }_{AB})}^{2}}\\  &  & \times \,\{1-{e}^{-{\alpha }^{2}{(2\sqrt{{R}_{4}}\sqrt{{R}_{1}}+\sqrt{1-{R}_{4}}\sqrt{{R}_{2}}({\eta }_{AB}{\eta }_{BC}-1))}^{2}}\}{e}^{-{R}_{4}{R}_{2}{\alpha }^{2}{(1-{\eta }_{AB}{\eta }_{BC})}^{2}}\end{array}$$

Since the derivation of Eqs (49–52) is very lengthy, we do not reproduce all the steps of the process. However, the derivation can be evaluated in a smilar manner as shown in the previous section.
